# Epstein-Barr virus-transformed B-cells from a hypoxia model of the germinal center requires external unsaturated fatty acids

**DOI:** 10.1371/journal.ppat.1013694

**Published:** 2025-11-11

**Authors:** Larissa Havey, Haixi You, Huimin Xian, John M. Asara, Rui Guo

**Affiliations:** 1 Department of Molecular Biology and Microbiology, Tufts University, Boston, Massachusetts, United States of America; 2 Division of Signal Transduction, Beth Israel Deaconess Medical Center and Department of Medicine, Harvard Medical School, Boston, Massachusetts, United States of America; University of Wisconsin-Madison, UNITED STATES OF AMERICA

## Abstract

Epstein-Barr virus (EBV) drives over 200,000 cancer cases annually, including diffuse large B-cell lymphoma, Burkitt lymphoma, and classic Hodgkin lymphoma—malignancies that frequently originate from germinal centers (GCs), which are physiologically hypoxic (O_2 _< 1%). However, conventional transformation models are typically conducted under 21% O_2_—an artificial condition that fails to replicate the hypoxic GC microenvironment and may obscure critical metabolic vulnerabilities that are therapeutically targetable. Therefore, therapeutic targets identified under 21% O_2_ conditions may not fully translate to the hypoxic environment of lymphoid tissues, which could limit their effectiveness *in vivo*. To overcome these limitations, we developed an *ex vivo* model of EBV-driven B-cell transformation under 1% O_2_, mimicking GC hypoxia. Under 1% O_2_, EBV efficiently transformed primary human B-cells, inducing hallmark oncogenic programs and activating super-enhancers at key loci including MYC and IRF4. Multi-omic profiling revealed a distinct hypoxia-adapted metabolic state, characterized by suppressed fatty acid synthesis, enhanced glycolysis and glycerophospholipid metabolism, and increased triglyceride storage in lipid droplets. These adaptations alleviate lipotoxic stress and maintain redox balance but render transformed cells highly dependent on external unsaturated fatty acids. Inhibition of triglyceride synthesis using the DGAT1 inhibitor A922500 selectively impaired proliferation and survival of EBV-transformed B-cells under GC-like hypoxia. These findings define key metabolic dependencies shaped by the hypoxic GC microenvironment and establish a physiologically relevant platform for studying EBV-driven B-cell transformation. Our work highlights the importance of modeling physiological oxygen tension and suggests that targeting lipid uptake and storage pathways may offer new therapeutic opportunities for halting EBV transformation with hypoxic tissue niches.

## Author summary

Epstein-Barr virus (EBV) is a common virus that silently persists in most people but can drive the development of aggressive B-cell cancers in individuals with weakened immune systems. Many of these cancers arise from germinal centers—specialized regions in lymphoid tissues that naturally have very low oxygen levels. However, most laboratory models of EBV infection are studied under atmospheric oxygen, which does not reflect the low-oxygen conditions found in these tissues. In this study, we developed a new model of EBV-driven B-cell transformation under physiological hypoxia to mimic the germinal center environment. We found that EBV-infected B-cells adapt their metabolism under low oxygen by suppressing fatty acid synthesis and relying instead on external unsaturated fatty acids for survival and growth. These cells also store excess saturated fatty acids in lipid droplets to avoid toxic stress. When we blocked the cells’ ability to store or use these lipids, their survival under hypoxia was significantly impaired. Our work shows that oxygen levels dramatically shape the metabolic requirements of EBV-transformed cells and highlights new opportunities for targeting virus-associated cancers in low-oxygen environments.

## Introduction

Epstein-Barr Virus (EBV) is a gammaherpesvirus that can transform human naïve B-cells into lymphoblastoid cell lines (LCLs) in culture. It infects approximately 90% of adults globally [1], and is implicated in various malignancies, including B-cell lymphomas like Diffuse Large B-cell Lymphomas (DLBCL), Burkitt Lymphomas (BL), and classic Hodgkin Lymphomas (cHL), as well as epithelial cancers such as nasopharyngeal and gastric carcinomas [2–7]. Due to its transformative capability, immunocompromised individuals, particularly those with HIV or undergoing post-transplant immunosuppressive treatments, are at an increased risk for developing these EBV-associated lymphoproliferative diseases and lymphomas [8,9].

EBV encodes Epstein-Barr virus nucleus antigens (EBNAs) and latent membrane proteins (LMPs) to transform B-cells. During the initial pre-latency phase, EBV expresses the Epstein-Barr virus nucleus antigen 2 (EBNA2) and EBNA-leader protein (EBNA-LP) [10–14]. EBNA2 transactivates c-MYC, a proto-oncogene by assembling a super-enhancer upstream of c-MYC gene loci [15,16]. EBNA2 and c-MYC transcriptionally remodel B-cell metabolism including aerobic glycolysis, oxidative phosphorylation (OXPHOS), mitochondrial one-carbon metabolism, mevalonate and fatty acid biosynthesis prior to the first mitosis, which metabolically prepares B-cells for upcoming hyperproliferation stages [14,17,18]. Starting from day 4 post-infection (DPI) the virus processes into the Latency IIb program. This stage is marked by the expression of all six EBNA proteins (EBNA1, 2, LP, 3A, 3B, and 3C) and BL-like hyperproliferation. Around 7 DPI, EBNA2 activates the bidirectional promotor of the latent membrane proteins 1 and 2 (LMP1 and LMP2) that take over the transformation processes that simulate key growth pathways in B-cells [19]. LMP1 functions as a constitutive CD40 receptor analog, activating NF-κB, JAK/STAT, and PI3K/AKT pathways to promote cell survival and proliferation. LMP2A mimics activated B-cell receptor signaling, maintaining latency and supporting cell growth by modulating PI3K/AKT and MAPK pathways [20,21].

**Fig 1:**
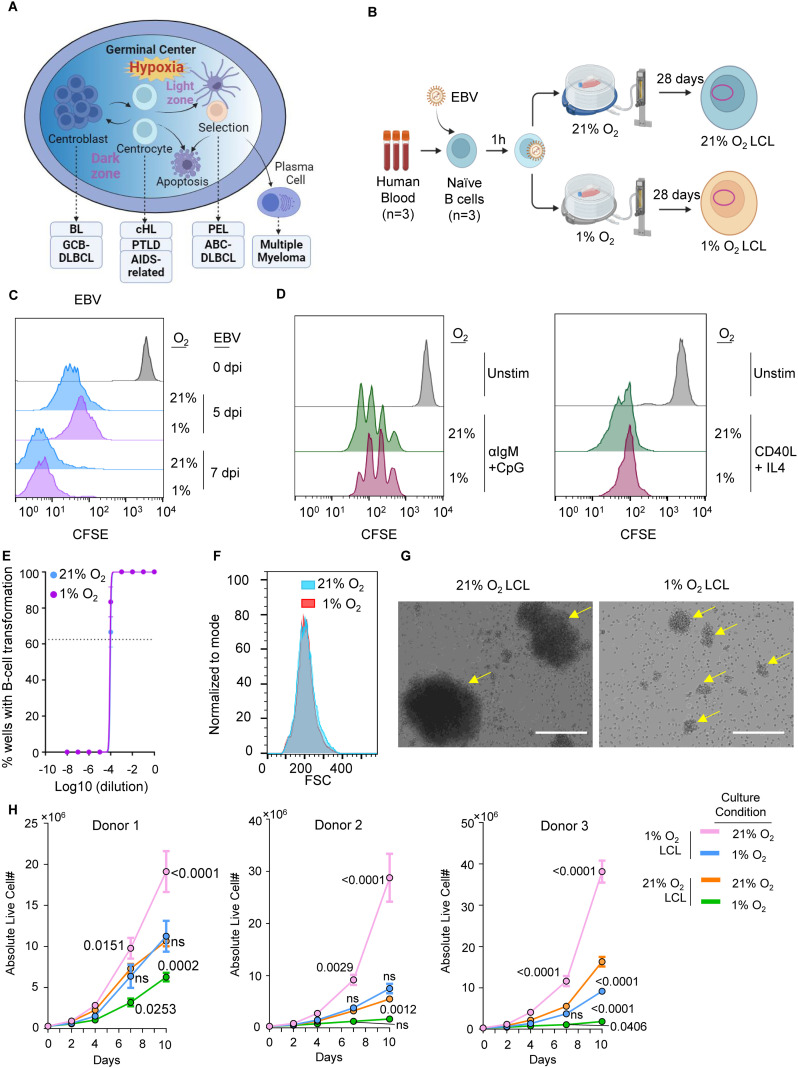
EBV transforms human primary B-cells under 1% O_2_. A. A schematic picture showing the GC origin of EBV-associated lymphomas. GC represents a physiologically relevant hypoxic microenvironment. BL, Burkitt Lymphoma; GCB-DLBCL, Germinal Center B-cell-like Diffuse Large B-cell Lymphoma; cHL,Classical Hodgkin Lymphoma; PTLD, Post-Transplant Lymphoproliferative Disorder; AIDS-related – AIDS-related Lymphoma; PEL, Primary Effusion Lymphoma; ABC-DLBCL, Activated B-cell-like Diffuse Large B-cell Lymphoma. This figure was created using biorender. B. A schematic picture of the new *ex vivo* model of EBV B-cell transformation under hypoxia. C. CFSE staining analysis of human primary B-cells transformed by EBV at a MOI of 0.1 under 1% or 21% O_2_ for 5 or 7 days. This is a representative FACS histogram plot from n = 3 independent donor. Only live cells were included in the analysis based on FSC/SSC gating to exclude dead cells and debris. D. CFSE staining analysis of human primary B-cells stimulated by a combination of 1µg/mL anti-human IgM IgG and 0.5 µM CpG or a combination of 50ng/mL CD40L and 20ng/mL IL-4 under 1% or 21% O_2_ for 5 days. This is a representative FACS histogram plot from n = 3 independent donor. Only live cells were included in the analysis based on FSC/SSC gating to exclude dead cells and debris. E. EBV transformation assays of primary human B-cells grown in 1% versus 21% O_2_. Shown are fitted non-linear regression curves with means ± SEM from n = 3 independent donor. F. Forward scatter (FSC) analysis of 1% or 21% O_2_ LCLs. This is a representative FACS histogram plot from n = 3 independent donor. G. Representative microscopic picture of 1% or 21% O_2_ LCLs from n = 3 independent donor. Arrows indicate the clumps in the LCL culture. Scale bar, 200 µM. H. Growth curve of 1% O₂ LCLs and 21% O₂ LCLs. Each LCL was split into two flasks, with one incubated at 1% O₂ and the other at 21% O₂. Mean + /- SEM values are from n = 3 experiments from 3 independent donor. P values were determined by using using two-way ANOVA with Dunnett’s post hoc test, comparing each group to the 21% O₂ LCL cultured under 21% O₂ conditions. LCLs derived from different donors are plotted separately.

The germinal center (GC) model of persistent EBV infection suggests that EBV-infected cells mirror the developmental pathway of uninfected B-cells as they differentiate into memory B-cells [22].The GC is composed of a dark zone, where B-cells called centroblasts proliferate and undergo somatic hypermutation, and a light zone, composed of non-dividing B-cells called centrocytes [23]. After GC entry, EBV-infected B-cells may transition from Latency III to Latency II or Latency I. This transition reflects a gradual reduction in viral gene expression, helping the infected cells evade immune detection. Specifically, EBV-infected B-cells transitioning to the memory B-cell phenotype often establish Latency I, characterized by the exclusive expression of EBNA1 [7]. Interestingly, most EBV-associated B-cell lymphomas are of GC B-cell origin [24] (Fig 1A), indicating that the lymphoid GC microenvironment might play a previously unrecognized role in EBV-driven tumorigenesis.

Oxygen tension naturally decreases across the human body, starting from arterial blood (~13%), moving through vital organs (~6%; including the brain, liver, and lungs), and reaching hypoxia in bone marrow and secondary lymphoid tissues (<3%; notably in lymph nodes) [25,26]. Remarkably, GCs in secondary lymphoid organs show oxygen tensions beneath 1%, as has been precisely determined using hypoxyprobe labeling, which enables the sensitive visualization of hypoxia in situ when oxygen levels fall below 1% [27,28]. Studies found that hypoxia strongly impacts B-cell activation in the GC light zone, significantly influencing B-cell development [29]. *In vivo* CRISPR screens highlighted that the GC hypoxic microenvironment can shape the function of T follicular helper cells and B-cell fate decisions [30].

Similarly, because EBV co-opts many of the same signaling networks used by uninfected B-cells in the GC [7,31], the profound hypoxia that shapes normal B-cell activation may also be integral to viral-driven transformation and tumorigenesis. Traditional *ex vivo* transformation models have provided valuable insights into EBV biology [24,31,32], but fail to replicate critical aspects of the hypoxic GC microenvironment. Particularly, these models may overlook the extensive metabolic reprogramming required for B-cell transformation under hypoxic conditions. Nevertheless, the metabolic pathways that are rewired under hypoxia may represent key vulnerabilities in EBV transformation or EBV-associated cancers. Investigating these hypoxia-driven metabolic pathways could provide new therapeutic targets for disrupting EBV-mediated oncogenesis.

To model EBV-driven B-cell transformation under physiologically relevant hypoxia, we developed a novel *ex vivo* transformation system under GC hypoxia. Integrated transcriptomic, metabolomic, and lipidomic analyses highlighted that hypoxically transformed LCLs suppress fatty acid synthesis, relying on extracellular unsaturated fatty acids for proliferation. Upregulated lipid droplet formation sequesters excess saturated lipids, mitigating lipotoxicity and supporting survival under physiologically relevant hypoxia, highlighting altered lipid supply as a potential vulnerability in EBV-associated malignancies.

## Results

### EBV transforms human primary B-cells under 1% O_2_ tension

In our new *ex vivo* transformation model (Fig 1B), freshly isolated CD19 + human resting B-cells are initially infected with the EBV strain B95.8 for one hour to standardize the initial viral entry across samples. Subsequently, these infected cells are divided equally into two groups: one group is cultured in a standard incubator with 21% O_2_, and the other under the hypoxic condition in an incubator with 1% O_2_. This setup mimics the natural infection process in immunocompromised patients who experience EBV lytic reactivation and viremia, where EBV-infected B-cells from peripheral blood migrate to the hypoxic environments of lymphoid organs. The cells are cultured for 28 days, during which time the EBV drives their transformation into LCLs [14,33,34]. A similar hypoxic condition has been used in Kaposi’s sarcoma-associated herpesvirus (KSHV) infection in SLK cells [35]. We observed that under 1% O_2_ conditions, EBV can comparatively transform human primary B-cells; CellTrace CFSE assay showed that EBV-driven B-cell proliferation under 1% O_2_ is comparable to that under 21% O_2_ during the first 7 days (Fig 1C). Similarly, combinations of mitogens—such as anti-human IgM/IgG to activate the B-cell receptor (BCR) pathway together with CpG to stimulate Toll-like receptor 9 (TLR9), or CD40 ligand (CD40L) with interleukin-4 (IL-4)—can induce B-cell proliferation under GC–like hypoxia (Fig 1D). While similar CFSE dilution profiles under both oxygen conditions suggested that EBV-infected B-cells proliferate at comparable rates, this did not exclude potential differences in transformation efficiency. To directly test this, we performed transformation unit assays using primary human B-cells under both 1% and 21% O₂. These assays revealed no difference in transformation efficiency, indicating that hypoxia does not impair EBV-mediated B-cell transformation (Fig 1E). Together, these results suggest that both EBV-driven transformation and mitogen-induced B-cell proliferation proceed efficiently under low-oxygen conditions.

By 28 DPI, LCLs transformed under 1% O₂ (hereafter referred to as 1% O₂ LCLs) exhibited comparable cell sizes to their normoxic counterparts, as assessed by forward scatter (FSC) in flow cytometry (Fig 1F). Notably, 1% O₂ LCLs formed clumps in culture, but to a much lesser extent in terms of size compared to the clumping observed in classical 21% O₂ LCLs [36,37] (Fig 1G). 1% O₂ LCLs demonstrated significantly higher proliferation rates than 21% O₂ LCLs when both were cultured under 1% O₂ conditions (Fig 1H), indicating superior adaptation to hypoxia that facilitates more robust growth. Strikingly, upon shifting to a 21% O₂ environment, 1% O₂ LCLs exhibited even greater proliferation rates than 21% O₂ LCLs under the same conditions (Fig 1H). Since 1% O₂ LCLs and 21% O₂ LCLs share the same genetic background, these findings suggest that EBV establishes a specialized transformation program tailored to the hypoxic GC microenvironment for enhanced proliferation and survival. Of note, this growth advantage was consistently observed across 1% O_2_ LCLs transformed from different donors (Fig 1H).

### Hypoxia has little impact on EBV oncogene expression and super-enhancer establishment

EBV employs a precisely controlled program to regulate B-cell transformation [10]. Upon infection, the EBV genome rapidly becomes chromatinized, expressing viral oncogenes and activating host regulatory networks critical for cell survival and proliferation [38]. To examine how oxygen availability influences key regulators of this transformative process, we assessed EBV oncogene expression in newly infected B-cells under 21% O₂ and 1% O₂ conditions.

**Fig 2:**
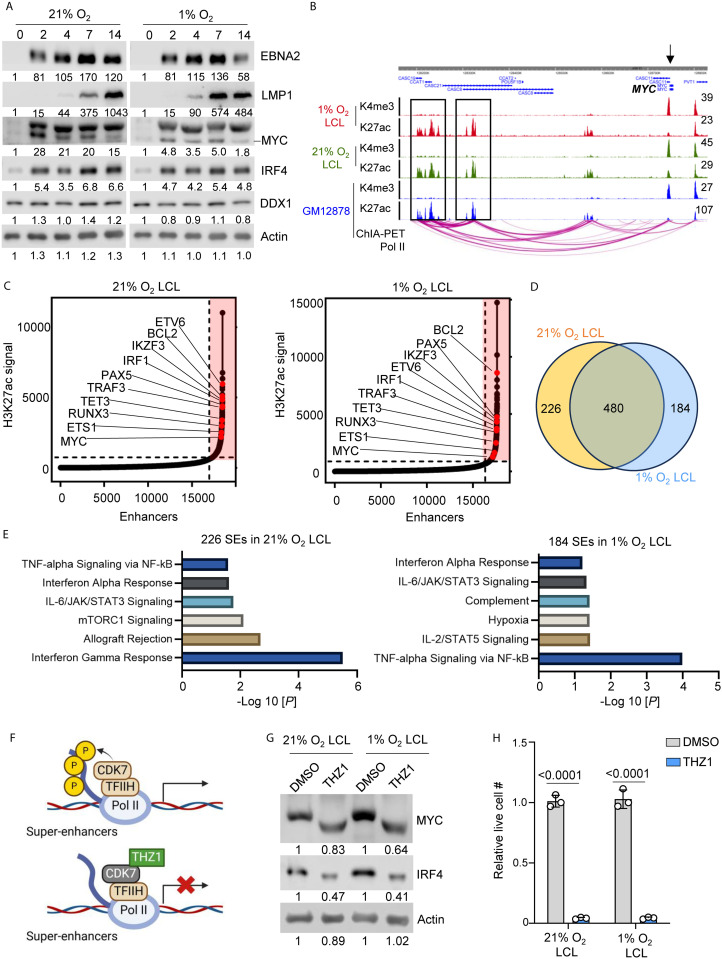
Hypoxia has minimal effect on EBV oncogene expression and the establishment of super-enhancers. A. Immunoblot analysis for indicated proteins in whole cell lysates (WCL) in EBV newly transformed human primary B-cells, collected at indicated days post-infection in either 1% or 21% O_2_. This is a representative experiment from donor 1. Analyses from another donor is in S1A Fig. B. H3K27ac and H3K4me3 ChIP-seq tracks from 1% O_2_, 21% O_2_, or GM12878 LCL, and GM12878 ChIA-PET Pol ll tracks are shown. The black arrow indicates MYC loci. Black boxes indicate SEs. These are the representative tracks from donor 1 LCLs. C. Enhancers are ranked by their H3K27ac ChIP-seq signals in either 1% or 21% O_2_ LCLs. The inflection point on the plotted curve was then selected as the cutoff to separate SEs from typical enhancers. SE associated genes are indicated in red box, selected of which are linked to their direct target genes by H3K27ac ChIP. D. Venn diagram analysis of SE-associated genes in 1% or 21% O_2_ LCLs. E. GSEA analysis of SEs specifically associated with 21% or 1% O_2_ LCLs. F. A schematic picture showing THZ1 inhibits CDK7 at SEs to suppress SE target gene expression. G. Immunoblot analysis of MYC, IRF4, and Actin from 1% or 21% O_2_ LCLs treated with DMSO or 100nM THZ1 for 48 h. Representative blot of n = 3 replicates from two independent donors shown. H. Relative live cell number in 1% or 21% O_2_ LCL treated with DMSO or 100 nM THZ1 for 4 days. Mean + /- SD values were from n = 3 experiments from two independent donors. P-values were calculated by two-way ANOVA with Sidak’s multiple comparisons test.

EBNA2, an essential transcription factor for EBV-driven B-cell outgrowth [39], exhibited a similar expression pattern at 2 DPI across both oxygen conditions, suggesting that its activation is largely oxygen-independent (Figs 2A and S1A). A major function of EBNA2 is the transactivation of MYC, a key proto-oncogene that regulates cell metabolism [40–43]. Together, EBNA2 and MYC orchestrate B-cell metabolic reprogramming to fuel transformation [17,44]. Consistent with EBNA2’s oxygen-independent expression, we observed that MYC expression peaked at 2 DPI in both 1% and 21% O₂ conditions; however, its relative expression level at 2 DPI was notably lower under hypoxia (Figs 2A and S1A). Interestingly, we observed an accelerated induction of LMP1 under 1% O_2_, occurring earlier than its 21% O_2_ counterpart, a trend that was consistently observed across different donors (Figs 2A and S1A). The earlier induction of LMP1 under hypoxia may be linked to the observed reduction in MYC expression (Figs 2A and S1A), as previous findings indicate that c-MYC represses LMP1 transcription in EBV-infected lymphoma cells and LCL models [12].

IRF4 is a key transcription factor in B-cell differentiation [45] and it plays a key role in maintaining EBV latency and B-cell survival [46,47]. In EBV-infected B-cells, IRF4 and BATF jointly repress tumor suppressors like PRDM1 and BCL2L11, thereby promoting transformation [47]. Given its role in EBV transformation, we assessed its expression under 1% and 21% O_2_ conditions. Our analysis revealed that IRF4 was induced under both 1% and 21% O_2_ conditions without notable differences (Figs 2A and S1A).

Super-enhancers (SEs) are clusters of highly active enhancers that regulate key genes driving cell identity and malignancy [48,49]. Compared with typical enhancers, SEs have larger size, higher transcription factor occupancy, and greater sensitivity to perturbation [48,49]. In LCLs, chromatin immunoprecipitation followed by sequencing (ChIP-seq), identified that EBNA2, LMP1, and NF-κB-driven SEs co-activate the expression of proto-oncogenes such as MYC and IRF4 [50]. Interestingly, hypoxia is known to influence SE activity through hypoxia-inducible factor (HIF)-mediated transcriptional regulation [51], but its impact on EBV-driven SEs remains unexplored. To address this, we performed H3K27ac ChIP-seq (to profile SE activity) and H3K4me3 ChIP-seq (to analyze promoter activity) in 1% or 21% O₂ LCLs. Our heatmap analysis revealed highly similar H3K4me3 and H3K27ac enrichment patterns between 1% and 21% O_2_ LCLs across transcription start sites (TSS) and transcription end sites (TES) (S1B Fig). We further examined key EBV-driven SE targets, including MYC, IRF4, and BCL2, and found no notable differences in their peak shapes and intensities between 1% and 21% LCLs, also with profiles comparable to those observed in GM12878 LCLs (Figs 2B and S1C). To assess the EBV-driven SEs in 1% and 21% O_2_ LCLs, we performed Homer SE analysis using H3K27ac ChIP-seq data. SE-associated genes were ranked based on H3K27ac signal intensity, revealing a highly similar SE landscape in 1% and 21% O₂ LCLs (Fig 2C and S1 Table). Key factors driving LCL survival and transformation, including *MYC*, ETS proto-oncogene 1 (*ETS1*), RUNX family transcription factor 3 (*RUNX3*), tet methylcytosine dioxygenase 3 (*TET3*), TNF receptor-associated factor 3 (*TRAF3*), interferon regulatory factor 1 (*IRF1*), IKAROS family zinc finger 3 (*IKZF3*), paired box 5 (*PAX5*), and BCL2 apoptosis regulator (*BCL2*), were consistently associated with SEs under both conditions (Fig 2C). Notably, the Venn diagram analysis showed substantial overlap in SE-associated genes between 1% and 21% O_2_ LCLs, with 480 shared SE-associated genes (Fig 2D). However, we also identified 226 SE-associated genes unique to normoxia and 184 unique to hypoxia (Fig 2D). Gene set enrichment analysis (GSEA) of these non-overlapping sets revealed distinct biology: SEs uniquely induced in 1% O_2_ LCLs were enriched in TNF-α signaling via NF-κB and hypoxia-related pathways, whereas 21% O_2_ LCL-unique SEs were enriched for interferon-γ response and allograft rejection pathways (Fig 2E).

Given these observations, we next investigated whether EBV-driven SEs remain viable therapeutic targets under hypoxic conditions [15]. SEs drive high-level transcription of key oncogenes by recruiting transcriptional machinery, including cyclin-dependent kinase 7 (CDK7), a critical component of the transcription initiation complex TFIIH [52]. As CDK7 phosphorylates RNA polymerase II, it facilitates transcriptional pause release and elongation, making SE-driven genes particularly dependent on its activity [53]. THZ1, a selective CDK7 inhibitor, disrupts this process by blocking transcriptional activation at SE-regulated loci, leading to preferential suppression of oncogenes with SE dependency [52] (Fig 2F). Accordingly, treatment with THZ1 effectively downregulated MYC and IRF4 expression (Fig 2G) and induced a significant growth defect in LCLs cultured under both 1% and 21% O₂ conditions (Fig 2H). We also noted mobility shifts of MYC and IRF4 in THZ1-treated LCLs, which were reproducible across replicates (Fig 2H). Collectively, our data indicates that EBV-driven oncogene expression and SEs are resilient to hypoxic conditions, ensuring sustained LCL growth and survival across varying oxygen levels.

### RNA-seq reveals hypoxia-specific transcriptomic adaptations in 1% O_2_ LCLs

**Fig 3:**
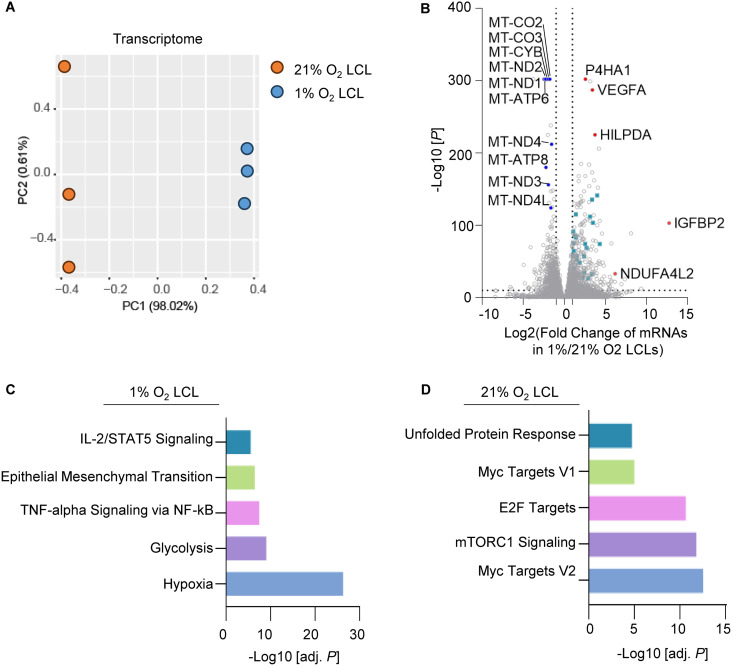
RNA-seq Reveals Hypoxia-Specific Transcriptomic Adaptations in 1% O_2_ LCLs. A. PCA analysis of transcriptome identified by RNAseq in 1% O_2_ LCLs (blue) or 21% O_2_ LCLs (orange), across n = 3 biological replicates using the LCLs derived from donor 1. B. Volcano plot visualization of -log_10_ (p-value statistical significance) vs log_2_ (mRNA abundance foldchange) from triplicate RNAseq analysis of 1% vs 21% O_2_ LCLs. Cyan, genes related to glycolysis. Common hypoxia-responsive genes are highlighted in red. C. Enrichr pathway analysis of gene sets significantly upregulated in 1% O_2_ LCLs. Shown are the -log_10_ (adjusted p-values) from Enrich analysis of triplicate RNAseq datasets. D. Enrichr pathway analysis of gene sets significantly upregulated in 21% O_2_ LCLs. Shown are the -log_10_ (adjusted p-values) from Enrich analysis of triplicate RNAseq datasets.

To explore the transcriptional changes associated with hypoxia during EBV-mediated B-cell transformation, we first performed RNA sequencing (RNA-seq) on 1% O₂ or 21% O₂ LCLs derived from donor 1. Principal component analysis (PCA) of the transcriptomes from donor 1–derived LCLs cultured under hypoxic (1% O₂) or normoxic (21% O₂) conditions revealed clear segregation of samples according to oxygen tension, with PC1 accounting for 98.02% of the total variance (Fig 3A). The top 20 genes contributing to PC1 are listed in S2 Table. To assess the reproducibility of this hypoxia-associated signature, RNA-seq was repeated using 1% or 21% O_2_ LCLs from three independent donors. Consistent expression patterns of the PC1-associated genes were observed across all donors, confirming that hypoxia drives a robust and conserved transcriptional program in EBV-transformed B-cells from different donors (S2A Fig).

Differentially expressed gene (DEGs) analysis revealed a significant number of hypoxia-induced genes with p-values <0.05 and fold change >2 (Fig 3B, S2 Table). Notably, hallmark hypoxia-responsive genes such as prolyl 4-hydroxylase subunit alpha 1 (*P4HA1*), vascular endothelial growth factor A (*VEGFA*), and hypoxia-inducible lipid droplet-associated protein (*HILPDA*) showed marked upregulation under 1% O₂ conditions. Additional genes, including NDUFA4 mitochondrial complex associated like 2 (*NDUFA4L2*), which downregulates mitochondrial OXPHOS activity to reduce oxidative stress, optimizing survival in low-oxygen environments [54], were also upregulated (Fig 3B). Conversely, mitochondrial DNA encoded genes, including *MT-ND* (Complex I), *MT-CYB* (Complex III), *MT-CO* (Complex IV), and *MT-ATP* (ATP synthase), were significantly downregulated under hypoxia, suggesting a suppression of OXPHOS (Fig 3B). Of note, glycolytic genes (highlighted in cyan in Fig 3B) were prominently upregulated in 1% O₂ LCLs.

Gene set enrichment analysis revealed pathways specific to the hypoxic condition in 1% O_2_ LCL. Terms such as glycolysis, epithelial-mesenchymal transition, and IL-2/STAT5 signaling were significantly enriched from upregulated DEGs under 1% O₂. The activation of glycolysis pathways aligns with the known metabolic switch to anaerobic energy production under hypoxia [55] (Fig 3C). In contrast, 21% O_2_ LCLs exhibited enrichment in pathways related to the unfolded protein response, MYC signaling, and mTORC1 activity, highlighting metabolic and signaling processes that are more prominent under oxygen-rich environments (Fig 3D).

To gain further insights into how 1% vs 21% O_2_ impacts EBV-driven B-cell metabolism, we next analyzed metabolic gene responses. DEGs upregulated in 1% O₂ LCLs were filtered within this metabolic gene set [56]. Subsequently, STRING, a protein-protein interaction networks functional enrichment analysis, was conducted using metabolic DEGs to depict the metabolic network specific to the 1% O_2_ LCLs (S2B Fig). Our analysis highlights a central role of glycolysis, with key genes such as aldolase, fructose-bisphosphate A (*ALDOA*), hexokinase 2 (*HK2*), 6-phosphofructo-2-kinase/fructose-2,6-biphosphatase 3 (*PFKFB3*), and lactate dehydrogenase A (*LDHA*) forming highly connected hubs, reflecting a metabolic shift toward anaerobic glycolysis as the primary pathway for ATP production. Notably, *HK2* and *PFKFB3* act as critical regulatory nodes linking upstream hypoxia-induced signaling to downstream glycolytic processes [57,58].

We found upregulation of key lipid metabolic regulators, including insulin-induced gene 2 (*INSIG2*) and sterol regulatory element-binding transcription factor 1 (*SREBF1*), which coordinate lipid biosynthesis and lipid droplet formation [59] (S2 Fig). SREBF1 is a master regulator of lipid metabolism [59,60], ensuring adequate lipid production for membrane biogenesis and energy storage [59]. INSIG2 acts as a crucial regulator of SREBF1 activation, controlling its release from the endoplasmic reticulum (ER) in response to lipid levels [61]. When cellular lipid stores are sufficient, INSIG2 prevents SREBF1 activation, thereby reducing lipid synthesis [61].

Additionally, genes involved in branched-chain amino acid (BCAA) catabolism, such as branched chain aminotransferase 1 (*BCAT1*), connect glycolysis with anaplerosis, maintaining TCA cycle activity under oxygen limitation (S2B Fig). These interactions highlight the metabolic flexibility of 1% O_2_ LCLs, which may utilize both carbohydrates and amino acids to sustain growth and adapt to fluctuating environmental conditions. Together, these metabolic adaptations underscore the ability of EBV-transformed LCLs to thrive in hypoxic environments, exploiting these niches to sustain growth and survival.

### Metabolic adaptations highlight glycolysis and redox shifts in 1% O_2_ LCLs

**Fig 4:**
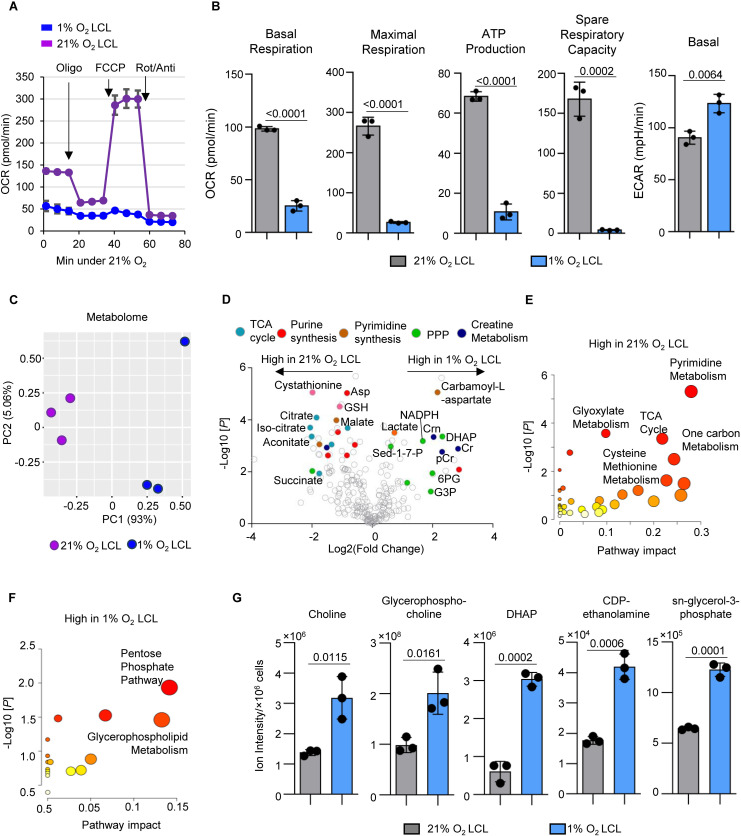
Hypoxia-Driven Metabolic Shifts Reshape Energy and Redox Balance in EBV-Transformed LCLs. A. Mitochondrial stress test of 1% or 21% O_2_ LCLs using a Seahorse analyzer. Both cells were seeded at 3 × 10^5^/mL. Mean + /- SD values were from n = 3 biological replicates using LCLs derived from donor 1.B. Seahorse OCR linked to basal respiration, maximal respiration, and ATP production and basal ECAR in 1% or 21% O_2_ LCLs. Mean + /- SD values were from n = 3 biological replicates. P-values were calculated using an unpaired Student’s t-test. C. PCA of metabolome identified by LC/MS metabolomic analysis in 1% O_2_ LCLs (blue) or 21% O_2_ LCLs (purple), across n = 3 biological replicates using LCLs derived from donor 1. D. Volcano plot visualization of -Log_10_ (p-value statistical significance) vs Log_2_ (metabolite abundance foldchange) from triplicate metabolomic analysis of 1% vs 21% O_2_ LCLs. E. Metabolic pathway analysis highlighting pathways significantly upregulated in 21% O_2_ LCLs. The x-axis shows pathway impact values from MetaboAnalyst 3.0 topological analysis; the y-axis shows -log_10_ of P-value from pathway enrichment analysis. F. Metabolic pathway analysis highlighting pathways significantly upregulated in 1% O_2_ LCLs. The x-axis shows pathway impact values from MetaboAnalyst 3.0 topological analysis; the y-axis shows -log_10_ of P-value from pathway enrichment analysis. G. Bar chart analysis of ion intensity of intermediates of glycerophospholipid metabolism in 1% or 21% O_2_ LCLs. Mean + /- SD values were from n = 3 biological replicates using LCLs derived from donor 1. P-values were calculated using an unpaired Student’s t-test.

To assess mitochondrial activity, we performed a Seahorse analysis to measure the oxygen consumption rate (OCR) in 1% and 21% O₂ LCLs. 1% O₂ LCLs exhibited significantly lower basal and maximal respiration, ATP production, and spare respiratory capacity (Fig 4A-B). Notably, this suppression of mitochondrial respiration is not due to reduced mitochondrial biogenesis; paradoxically, MitoTracker Green staining revealed significantly higher mitochondrial content in 1% O₂ LCLs (S3A Fig), suggesting a potential compensatory response, as previously reported [62–64]. Consistent with RNA-seq data, the extracellular acidification rate (ECAR) was significantly elevated in 1% O₂ LCLs, indicating a shift toward glycolysis (Fig 4B).

We next performed liquid chromatography-mass spectrometry (LC/MS) analysis to compare metabolome in 1% and 21% O₂ LCLs. PCA analysis revealed distinct metabolic shifts, with PC1 accounting for 93% of the variance, demonstrating clear clustering based on oxygen conditions (Fig 4C). 21% O₂ LCLs displayed higher levels of TCA cycle intermediates, reflecting their greater reliance on mitochondrial oxidative metabolism (Fig 4D-E and S3 Table). Notably, key metabolites in one-carbon metabolism and pyrimidine synthesis were enriched, suggesting enhanced nucleotide biosynthesis to support rapid proliferation. These metabolic features are consistent with the other conventional EBV transformation models, where oxygen availability enables mitochondrial ATP generation and biosynthetic pathways necessary for biomass accumulation [14,17].

In contrast, 1% O₂ LCLs exhibited increased levels of glycolytic and pentose phosphate pathway (PPP) intermediates (Fig 4D). We observed a significantly elevated NADPH/NADP⁺ ratio in 1% O₂ LCLs, suggesting a shift in redox balance to counteract hypoxia-induced reactive oxygen species (ROS) (S3B Fig). While heightened PPP activity is one possible contributor [65,66], treatment with 6-aminonicotinamide (6-AN), a glucose-6-phosphate dehydrogenase (G6PD) inhibitor, had little selective inhibition on 1% O₂ LCL growth (S3C Fig). This finding suggests that these cells engage compensatory NADPH-generating routes, such as malic enzyme (ME1)– and cytosolic isocitrate dehydrogenase (IDH1)–mediated NADPH generation, to sustain redox homeostasis under oxygen limitation [67,68]. The engagement of multiple NADPH-producing nodes likely reflects a broader metabolic plasticity program driven by EBV, ensuring redox resilience despite reduced mitochondrial oxidative metabolism under hypoxia.

Notably, the NADH/NAD⁺ ratio remained unchanged. Given the increased lactate production and the elevation of *LDHA*, this homeostasis may be maintained through efficient NAD⁺ regeneration via fermentation. Additionally, creatine metabolism was significantly upregulated in 1% O_2_ LCLs, consistent with its previously reported role as a rapid ATP buffer system during hypoxia [69,70] (Fig 4D).

Of interest, glycerophospholipid metabolism was elevated in 1% O_2_ LCLs (Fig 4F). 1% O₂ LCLs exhibited markedly increased levels of choline, glycerophosphocholine, dihydroxyacetone phosphate (DHAP), CDP-ethanolamine, and sn-glycerol-3-phosphate compared to 21% O₂ LCLs (Fig 4G). These metabolites are essential intermediates in the biosynthesis of phosphatidylcholine (PC) and phosphatidylethanolamine (PE), major components of cellular membranes. Notably, the elevation of DHAP and sn-glycerol-3-phosphate—key intermediates linking glycolysis to triglyceride (TG) synthesis—suggests a broader metabolic shift beyond membrane remodeling.

### Hypoxia induces triglyceride storage and lipid droplet formation in 1% O₂ LCLs

**Fig 5:**
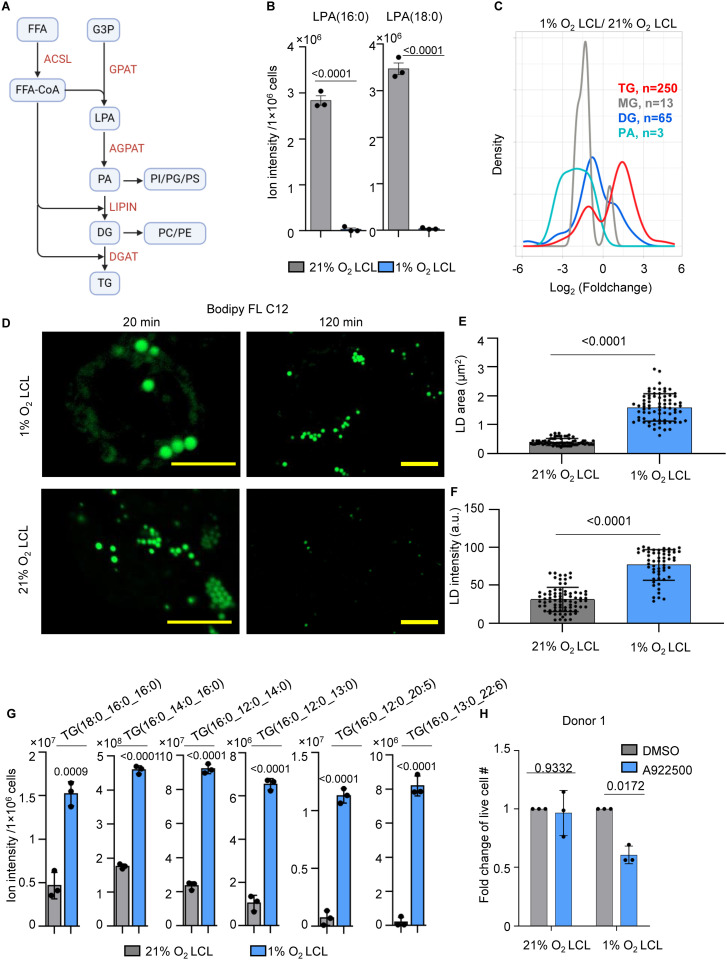
Hypoxia induces TG biosynthesis and lipid droplet formation in 1% O₂ LCLs. A. A schematic picture showing phospholipids and TG biosynthetic pathway. FFA, free fatty acids; FFA-CoA, free fatty acid coenzyme A; G3P, sn-glycerol-3-phosphate; LPA, lysophosphatidic acid; PA, phosphatidic acid; PI, phosphatidylinositol; PG, phosphatidylglycerol; PS, phosphatidylserine; DG, diacylglyceride; PC, phosphatidylcholine; PE, phosphatidylethanolamine. B. Bar chart analysis of ion intensity of indicated LPA species in 1% or 21% O_2_ LCLs. Mean + /- SD values were from n = 3 biological replicates using LCLs derived from donor 1. P-values were calculated using an unpaired Student’s t-test. C. Density plot analysis of log_2_ (lipid abundance foldchange) of indicated lipid species from triplicate lipidomic analysis of 1% vs 21% O_2_ LCLs. D. Confocal microscopic analysis of 1% vs 21% O_2_ LCLs treated with 10 µM Bodipy FL C12 for 20 min or 120 min. Representative of n = 3 experiments using LCLs derived from donors 1 and 2. Scale bar, 5 µm. E. LD area analysis was performed on 1% and 21% O₂ LCLs treated with 10 µM Bodipy FL C12 for 20 minutes. LD area was quantified using ImageJ from three randomly selected images, each containing 4 ~ 5 cells. F. LD fluorescent intensity was performed on 1% and 21% O₂ LCLs treated with 10 µM Bodipy FL C12 for 120 minutes. Bodipy FL C12 intensity was quantified using ImageJ from three randomly selected images, each containing 4 ~ 5 cells. G. Bar chart analysis of ion intensity of indicated TG species in 1% or 21% O_2_ LCLs. Mean + /- SD values were from n = 3 experiments using LCLs derived from donor 1. P-values were calculated using an unpaired Student’s t-test. H. Fold change of live cell number of 1% or 21% O_2_ LCL treated with DMSO or 10 µM of A922500, a DGAT1 inhibitor for 72 hours. Mean + /- SD values were from n = 3 experiments using LCLs derived from donor 1 (replicates using donor 2 LCLs are shown in S4C fig). P-values were determined using two-way ANOVA with Sidak’s multiple comparisons test.

We next investigated whether 1% O₂ LCLs rewire TG metabolism as an adaptive strategy to hypoxia. TG biosynthesis is a multi-step process, which plays a key role in hypoxia adaptation by sequestering toxic saturated lipids [71]. In this pathway, fatty acyl-CoA is first processed by glycerol-3-phosphate acyltransferase (GPAT) to form lysophosphatidic acid (LPA), which is further acylated by 1-acylglycerol-3-phosphate O-acyltransferase (AGPAT) to produce phosphatidic acid (PA). PA is then dephosphorylated by phosphatidate phosphatase (LIPIN) to generate diacylglycerol (DG), which is finally converted into TG by diacylglycerol O-acyltransferase (DGAT) (Fig 5A).

Our lipidomic analysis revealed a significant depletion of lysophosphatidic acid (LPA) species, specifically LPA(16:0) and LPA(18:0), in 1% O₂ LCLs (Fig 5B), accompanied by a global accumulation of triglycerides (TG) (Fig 5C and S4 Table). This shift suggests a metabolic adaptation favoring TG synthesis under hypoxia in 1% O_2_ LCLs. This reprogramming likely serves two primary functions: (1) redirecting saturated fatty acids away from membrane lipid synthesis to mitigate lipotoxic stress, particularly as stearyl desaturase 1 (SCD1) and fatty acid oxidation (FAO) are inhibited due to limited O₂ availability, and (2) increasing lipid storage capacity to buffer against energy fluctuations [71–73]. Notably, Luftig lab recently demonstrated the critical roles of SCD1 and fatty acid desaturase 2 (FADS2) in EBV transformed LCLs [74].

TGs are stored in lipid droplets (LDs). RNA-seq analysis revealed significant upregulation of perilipin 2 (*PLIN2*) and perilipin 3 (*PLIN3*) in 1% O₂ LCLs, while other *PLIN*s remained barely expressed (S4A Fig). PLIN2 stabilizes LDs and promotes TG accumulation, while PLIN3 facilitates lipid storage and trafficking [75]. Additionally, the upregulation of very low-density lipoprotein receptor (*VLDLR*) suggests increased lipid uptake (S4A Fig). To visualize LDs, we treated 1% O₂ and 21% O₂ LCLs cells with Bodipy FL C12, a saturated lipid probe and performed live-cell confocal microscopy. After 20 min of labeling, LDs were clearly detected, with 1% O₂ LCLs containing significantly larger LDs compared with 21% O₂ LCLs, although the number of LDs appeared higher under normoxia (Fig 5D–E). Extending the labeling to 120 min led to diminished Bodipy fluorescence in 21% O₂ LCLs, but fluorescence persisted in 1% O₂ LCLs, suggesting that 1% O_2_ LCLs have enhanced capacity for lipid storage (Fig 5D and F).

Notably, TG species such as TG(16:0_16:0_16:0) and TG(18:0_16:0_16:0), composed entirely of saturated acyl chains, were significantly elevated in 1% O_2_ LCLs. Additionally, TGs incorporating both saturated and unsaturated fatty acids, including TG(16:0_12:0_20:5) and TG(16:0_13:0_22:6), were also significantly enriched (Fig 5G). This supports the hypothesis that 1% O₂ LCLs store saturated lipids in LDs as a protective strategy against lipotoxicity.

To further test this hypothesis, we treated 1% and 21% O₂ LCLs with A922500, a selective DGAT1 inhibitor, to block TG biosynthesis. A922500 treatment effectively inhibited LD biogenesis in 1% O_2_ LCLs, confirming its on-target effects (S4B Fig). Notably, A922500 selectively impaired the growth of 1% O₂ LCLs from multiple donors, while having minimal impact on 21% O₂ LCLs (Figs 5H and S4C). Moreover, A922500 treatment selectively increased Caspase 3/7 activity in 1% O₂ LCLs, further supporting the critical role of TG synthesis and LD formation in 1% O_2_ LCL survival (S4D Fig).

### Extracellular unsaturated fatty acids are essential for the survival of 1% O_2_ LCLs

**Fig 6:**
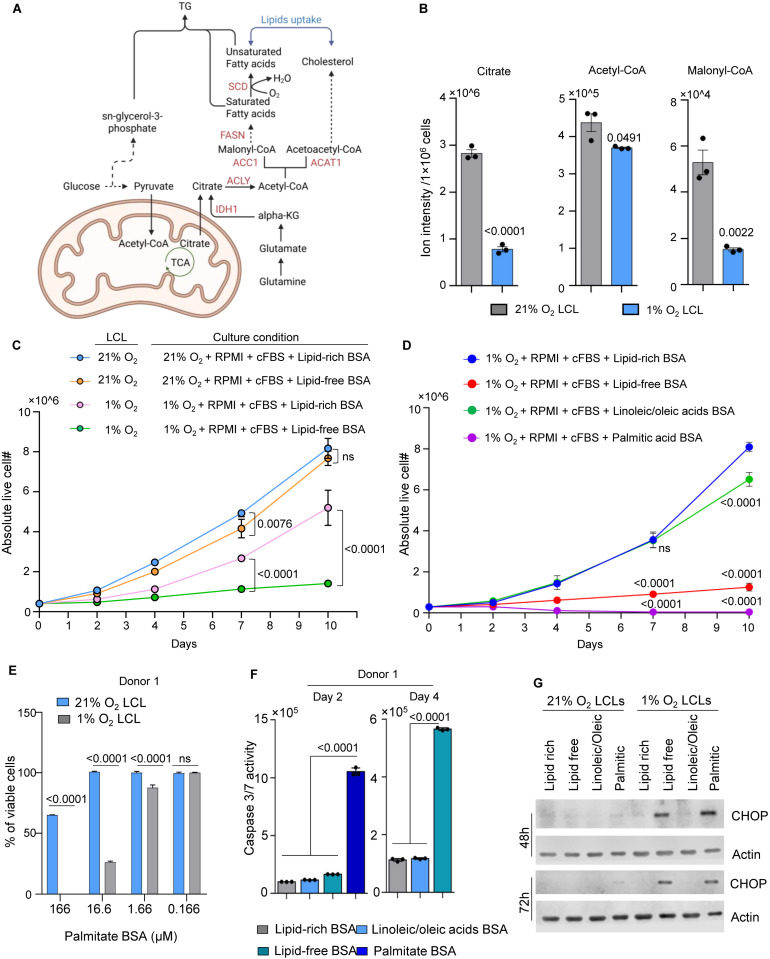
Extracellular unsaturated fatty acids are essential for the survival of 1% O_2_ LCLs. A. A schematic picture showing fatty acid synthesis and mevalonate pathway. This figure was created using biorender. B. Bar chart analysis of ion intensity of indicated metabolites in 1% or 21% O_2_ LCLs. Mean + /- SD values were from n = 3 experiments using donor 1 LCLs. P-values were calculated using an unpaired Student’s t-test. C. Growth curve of 1% O₂ LCLs and 21% O₂ LCLs cultured in indicated condition. Abbreviations in the culture condition: 21% O_2_, 21% O_2_ incubator; 1% O_2_, 1% O_2_ incubator; RPMI, RPMI-1640 media; cFBS, 10% charcoal-stripped FBS; Lipid-rich BSA, 1mg/mL; Lipid-free BSA, 1mg/mL. Mean + /- SD values are from n = 3 experiments using donor 1 LCLs. P-values were calculated using two-way ANOVA with Tukey’s multiple comparisons test. D. Growth curve of 1% O₂ LCLs cultured in indicated condition. LCLs derived from different donors are plotted separately in the extended fig. Abbreviations in the culture condition: 1% O_2_, 1% O_2_ incubator; RPMI, RPMI-1640 media; cFBS, 10% charcoal-stripped FBS; Lipid-rich BSA, 1mg/mL; Lipid-free BSA, 1mg/mL; Linoleic/oleic acids BSA, 1mg/mL; Palmitic acid BSA, 0.36 mg/mL. Mean + /- SD values are from n = 3 experiments using donor 1 LCLs (replicates using different donors are shown in S5B fig). P-values were calculated using two-way ANOVA with Dunnett’s multiple comparisons test, comparing each group to the 1% O₂ LCL cultured under lipid rich media. E. Palmitic acid dose–response assay in LCLs cultured under 21% O₂ or 1% O₂ conditions. Cells were treated with the indicated concentrations of palmitate–BSA conjugate and maintained under the specified oxygen conditions for 72 hours. Cell viability was assessed by Trypan Blue exclusion using an automated cell counter. Data represent mean ± SD from three independent experiments using LCLs derived from donors 2 and 3; replicate results using different donor-derived LCLs are shown in S5C Fig. F. Caspase 3/7 activity in 1% O_2_ LCLs cultured in the lipid free media repleted with lipid-rich, lipid-free, oleic/linoleic acid, or palmitic acid BSA as in D. Mean + /- SD values were from n = 3 experiments using donor 1 LCLs (replicates using different donors are in S5D Fig). P-values were calculated using one-way ANOVA with Holm-Sidak’s multiple comparisons test. G. Immunoblot analysis of CHOP and Actin in WCLs from 21% or 1% O_2_ LCLs cultured in the lipid free media repleted with lipid-rich, lipid-free, oleic/linoleic acid, or palmitic acid BSA as in D. Representative blots are shown from n = 3 experiments.

Previous research identified that mevalonate and fatty acid synthesis (FAS) pathways were amongst the most highly EBV induced [18]. Cholesterol and fatty acid biosynthesis begins with citrate, which is exported from mitochondria or synthesized in the cytosol via the reductive carboxylation of α-ketoglutarate (α-KG) catalyzed by isocitrate dehydrogenase 1 (IDH1). Citrate is converted to acetyl-CoA by ATP citrate lyase (ACLY). Acetyl-CoA is then carboxylated to malonyl-CoA by acetyl-CoA carboxylase (ACC1, encoded by *ACACA*), a rate-limiting enzyme in fatty acid synthesis. Malonyl-CoA and acetyl-CoA are subsequently utilized for fatty acid elongation, facilitated by fatty acid synthase (FASN) and related enzymes. For cholesterol biosynthesis, acetoacetyl-CoA, derived from acetyl-CoA, enters the mevalonate pathway, which produces key intermediates for cholesterol and other isoprenoid compounds essential for membrane structure and intracellular trafficking (Fig 6A).

Our RNA-seq analysis revealed significant downregulation of *ACLY*, *ACACA*, *FASN*, and *IDH1* in 1% O₂ LCLs compared to 21% O₂ LCLs (S5A Fig). Consistently, metabolomic analysis showed a marked reduction in key FAS intermediates, including citrate, acetyl-CoA, and malonyl-CoA, while sn-glycerol-3-phosphate, a precursor for triglycerides and phospholipids, was significantly elevated in 1% O₂ LCLs (Figs 4G and 6B). Newly synthesized saturated fatty acids can be cytotoxic under hypoxic conditions, particularly when desaturation (via SCD1) and FAO are suppressed. Therefore, the observed downregulation of FAS genes and metabolites in 1% O₂ LCLs likely reflects an adaptive response to limit lipotoxicity and maintain cellular homeostasis in the hypoxic environment.

The suppression of FAS in 1% O₂ LCLs suggests that these cells must obtain lipids from alternative sources to sustain membrane synthesis and energy storage during the rapid proliferation. We hypothesized that 1% O₂ LCLs rely on external fatty acids to meet their metabolic demands. To test this, we cultured 1% and 21% O₂ LCLs in lipid-rich or lipid-low media. While 21% O₂ LCLs proliferated regardless of external lipid availability, 1% O₂ LCLs exhibited significantly impaired growth in lipid-low conditions. Supplementation with lipid-rich bovine serum albumin (BSA conjugated with linoleic, oleic, palmitic, stearic acids) partially rescued their growth, underscoring their dependence on external lipids for survival under hypoxia (Fig 6C). Interestingly, linoleic/oleic acid BSA supplementation were sufficient to restore growth, whereas palmitate-BSA exacerbated cell death (Fig 6D). This metabolic dependency was consistently observed across 1% O₂ LCLs from three independent donors, suggesting a conserved adaptation to hypoxic stress (S5B Fig). Although at first glance this appears counterintuitive, given our model that 1% O₂ LCLs engage compensatory mechanisms to mitigate saturated fatty acid stress, these defenses are capacity-limited and critically constrained by the oxygen dependence of SCD1-mediated desaturation. Thus, under hypoxia, excess palmitate accumulates in toxic forms despite compensatory pathways such as lipid droplet sequestration. Consistent with this mechanism, palmitate reduced viability more strongly at 1% O₂ than at 21% O₂ at matched doses (Figs 6E and S5C). Furthermore, palmitate-BSA supplementation rapidly increased caspase 3/7 activity, indicating acute induction of apoptosis (Figs 6F and S5D). In contrast, fatty acid-free media triggered caspase 3/7 activation more gradually, suggesting a slower but progressive onset of apoptosis due to lipid deprivation (Figs 6F and S5D).

In general, external fatty acids can influence cell fitness through two nonexclusive mechanisms—(i) replenishing intracellular lipid pools that support membrane synthesis and ER homeostasis, and (ii) acting as GPCR ligands that activate lipid-sensing signaling pathways. When external fatty acids enter the cytosol, they are converted by acyl-CoA synthetase long-chain family members (ACSLs) into fatty acyl-CoA, which serves as a substrate for lipid metabolism and membrane biogenesis. To assess the contribution of this uptake–activation pathway, we treated 1% and 21% O₂ LCLs with Triacsin C, a pan-ACSL inhibitor [76]. Notably, 1% O₂ LCLs were more sensitive to Triacsin C across three different donors (S6 Fig), indicating a heightened reliance on ACSL-dependent lipid activation. However, the effect size was notably smaller than that observed upon extracellular unsaturated fatty acid depletion, suggesting the existence of compensatory lipid uptake/activation mechanisms. Indeed, our RNA-seq analysis revealed that, in addition to *LDLR*, *SLC27A1* (*FATP1*) was significantly upregulated in 1% O₂ LCLs, whereas *SLC27A4* (*FATP4*) was downregulated (S7 Fig, S2 Table). In contrast, *CD36*, a major scavenger receptor for long-chain fatty acid uptake in many cell types, was not detectably expressed under either 1% or 21% O_2_ LCLs (S7 Fig, S2 Table). FATP1 couples fatty acid transport with acyl-CoA synthetase activity to mediate long-chain fatty acid import and activation [77]. Its induction under hypoxia may compensate for suppressed *de novo* lipogenesis, sustaining fatty acid uptake and utilization when endogenous synthesis is limited. We therefore hypothesize that FATP1 serves as a key facilitator of lipid import under hypoxia, potentially buffering metabolic stress and supporting the increased extracellular lipid dependence of hypoxic LCLs. The redundant acyl-CoA synthetase activity of FATP1 may also account for the reduced impact of Triacsin C inhibition relative to lipid withdrawal.

It remains possible that fatty acid supplementation affects cell fitness not only through alteration of lipid pools but also via GPCR-mediated signaling. Saturated fatty acids such as palmitate can activate proinflammatory GPCRs (e.g., GPR84), whereas unsaturated fatty acids preferentially engage anti-inflammatory receptors (e.g., GPR120) [78,79]. However, RNA-seq analyses revealed negligible expression of both *GPR84* and *GPR120* in LCLs at either 21% or 1% O₂ (S7 Fig, S2 Table). Instead, our immunoblotting data indicates that linoleic/oleic fatty acids supplementation rescues hypoxic LCLs primarily by alleviating ER stress. We found that lipid deprivation at 1% O₂ induced CHOP expression, which was suppressed by linoleic/oleic acids but exacerbated by palmitic acids (Fig 6G). Thus, while GPCR contributions cannot be fully excluded, the dominant mechanism underlying the rescue of hypoxic LCLs by unsaturated fatty acids is the restoration of lipid homeostasis and mitigation of ER stress.

### Mevalonate pathway remains critical for protein prenylation in 1% O_2_ LCLs

RNAseq analysis of 1% and 21% O_2_ LCLs revealed that acetyl-CoA acetyltransferase 1 (*ACAT1*), an enzyme critical for the condensation of acetyl-CoA into acetoacetyl-CoA in the early steps of the mevalonate pathway, was significantly downregulated in 1% O₂ LCLs, indicating potential suppression of the pathway under hypoxia (Figs 6A and S8A). In contrast, the expression of low-density lipoprotein receptor (LDLR), a main cholesterol transporter, remained unchanged, suggesting that cholesterol uptake from extracellular sources may compensate for reduced cholesterol biosynthesis (S8A Fig). Consistent with this, metabolomics analysis showed that while total cholesterol levels were unaffected, there was a significant reduction in acetoacetyl-CoA, a precursor in the mevalonate pathway, in 1% O₂ LCLs. This supports the idea that external cholesterol uptake via LDLR maintains cellular cholesterol levels under hypoxic conditions (S8B Fig).

Protein prenylation is essential for EBV-transformed LCLs, with geranylgeranylation playing a key role in small GTPase activation, including Rab and Rho family members, which regulate intracellular trafficking, cytoskeletal dynamics, and signal transduction [18]. Geranylgeranyl pyrophosphate (GGPP), a crucial product of the EBV-induced mevalonate pathway, activates Rab13, facilitating LMP1 and LMP2A trafficking and signaling that are necessary for GM12878 LCL survival and proliferation [18]. To assess the role of this pathway in 1% O₂ LCLs, we treated cells with simvastatin, an HMG-CoA reductase (HMGCR) inhibitor, which blocked the mevalonate pathway (S8C Fig). Both 1% and 21% O₂ LCLs were highly sensitive to simvastatin, exhibiting significant growth defect, highlighting the conserved role of the mevalonate pathway in LCL survival (S8D Fig). Notably, supplementation with GGPP rescued the growth of 1% O₂ LCLs, consistent with previous findings in GM12878 LCLs [18] (S8E-G Fig). These results strongly suggest that the primary function of the mevalonate pathway in LCLs is to supply intermediates for protein prenylation, reinforcing the critical dependency of LCLs on geranylgeranylation to sustain EBV oncogenic signaling, regardless of oxygen levels.

### Hypoxia-driven metabolic rewiring is uniquely engaged by EBV during early infection

**Fig 7:**
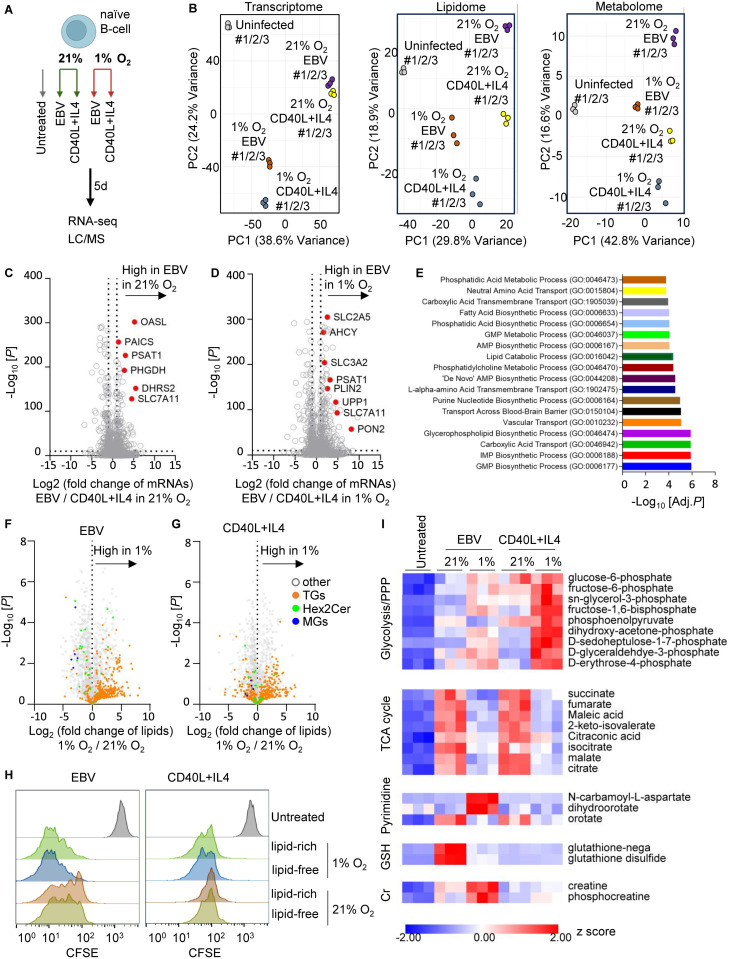
Hypoxia-driven metabolic rewiring is uniquely engaged by EBV during early infection. A. Freshly isolated human naïve B-cells were either infected with the EBV strain B95.8 at a multiplicity of infection (MOI) of 1 or stimulated with 50 ng/mL CD40L and 20 ng/mL IL-4. Cells were cultured under 21% O₂ or 1% O₂ conditions for 5 days and then harvested for multi-omics analyses. Untreated B-cells served as baseline control. This figure was created using biorender. B. PCA analysis of transcriptome, lipidome, and metabolome in cells described in A, across n = 3 biological replicates using the LCLs derived from donor 4.C. Volcano plot visualization of -Log_10_ (p-value) vs Log_2_ (mRNA foldchange) from triplicate transcriptomic analysis of EBV vs CD40L+IL4 under 21% O_2_. A curated metabolic gene list was used to filter the dataset to highlight the switch in metabolic pathways.D. Similar analysis as in (C), comparing EBV-infected and CD40L+IL-4–stimulated B-cells cultured under hypoxic (1% O₂) conditions.E. GO Biological Process analysis of metabolic genes significantly upregulated in EBV-infected B-cells cultured under hypoxic (1% O₂) conditions compared with CD40L+IL-4–stimulated cells at 1% O₂ (P < 0.001, log₂ fold change > 1).F. Volcano plot visualization of -Log_10_ (p-value) vs Log_2_ (lipids foldchange) from triplicate lipidomic analysis of EBV infected cells under 1% vs 21% O_2_. G. Volcano plot visualization of -Log_10_ (p-value) vs Log_2_ (lipids foldchange) from triplicate lipidomic analysis of CD40L+IL4 stimulated cells under 1% vs 21% O_2_. H. CFSE-based proliferation analysis of EBV-infected or CD40L+IL-4–stimulated naïve B-cells cultured under 21% or 1% O₂ conditions in lipid-rich or lipid-free media. Untreated B-cells were included as non-proliferating controls. Flow cytometry was performed at 5 days post infection or stimulation. Only live cells were included in the analysis based on FSC/SSC gating to exclude dead cells and debris. I. Heatmap showing the relative ion intensities (z-scores) of selected metabolites identified in the metabolomics analysis described in (A), highlighting pathways involved in glycolysis and the pentose phosphate pathway (PPP), the TCA cycle, pyrimidine synthesis, glutathione (GSH) metabolism, and creatine metabolism. Z-scores represent the deviation of each metabolite’s abundance from the mean across samples, expressed in units of standard deviation.

To distinguish EBV-specific metabolic responses from generic hypoxia adaptation, we compared untreated B-cells, EBV-infected B-cells, and CD40L+IL4-stimulated B-cells cultured under 21% or 1% O₂ (Fig 7A). Because CD40L+IL4-stimulated B-cells cannot be viably maintained beyond ~7–10 days, analyses were performed at 5 dpi, a stage-matched window that captures early remodeling before secondary selection effects. At this time point, both EBV- and CD40L+IL4-stimulated B-cells proliferated comparably under hypoxia (Fig 1D), ruling out differences in growth as a confounding factor.

PCA was performed on the transcriptome, lipidome, and metabolome to assess how EBV infection and CD40L + IL-4 stimulation reshape cell metabolism under 1% and 21% O₂ conditions (Fig 7B, S5 Table). Across all datasets, PC1 primarily separated activated or proliferating B-cells—both EBV-infected (purple, orange) and CD40L + IL-4–stimulated (yellow, blue)—from uninfected resting B-cells (gray), reflecting a shared activation state. In the transcriptome, hypoxia shifted both EBV- and CD40L + IL-4–stimulated cells along PC1 to a similar extent, indicating comparable transcriptional adaptation to low oxygen. However, PC2 captured subtle differences between the two activation states under hypoxia, suggesting minor divergence in oxygen-dependent transcriptional programs. In contrast, in the lipidome and metabolome, hypoxia caused more pronounced redistribution along both PC1 and PC2 in EBV-infected cells, indicating that EBV amplifies hypoxia-driven remodeling of lipid and small-molecule metabolism (Fig 7B).

Differential expression analysis of transcriptome identified a distinct cluster of hypoxia-inducible metabolic genes selectively upregulated by EBV, including solute carrier family 2 member 5 (*SLC2A5*), phosphoserine aminotransferase 1 (*PSAT1*), uridine phosphorylase 1 (*UPP1*), solute carrier family 7 member 11 (*SLC7A11*), and *PLIN2* (Fig 7C–D). These genes encode transporters and enzymes central to nucleotide biosynthesis, redox balance, and lipid droplet biogenesis, suggesting that EBV amplifies metabolic arms of the hypoxia response beyond those induced by cytokine activation. Furthermore, gene ontology enrichment revealed that EBV infection under 1% O_2_ exhibited more pronounced engagement of glycerophospholipid and pyrimidine biosynthesis pathways (Fig 7E).

Integration of transcriptomic and lipidomic profiles revealed tight correspondence between transcriptional induction of PLIN2 and the accumulation of TGs together with depletion of MGs under hypoxia (Fig 7F–G). These changes were substantially weaker in CD40L+IL4-stimulated B-cells. EBV-infected B-cells also exhibited pronounced remodeling of hexosylceramides (Hex2Cer), suggestive of selective modulation of sphingolipid pathways (Fig 7F–G). These observations suggest that EBV-driven transcriptional programs may reshape lipid metabolism to accommodate altered energy and redox demands under hypoxia.

To assess whether external lipids are already required for early proliferation driven by EBV or CD40L+IL4 under hypoxia, we performed a CFSE-based proliferation assay. Interestingly, both EBV-infected and CD40L+IL4-stimulated B-cells proliferated comparably under lipid-rich and lipid-free conditions at 1% O₂ (Fig 7H), indicating that newly infected or stimulated B-cells are not immediately dependent on external lipids for initial outgrowth. This contrasts with established LCLs, where hypoxia drives a strict requirement for external unsaturated fatty acids (Fig 6D). These results suggest a temporal model in which EBV-infected B-cells initiate unique lipid remodeling during the early hypoxic exposure, but full acquisition of lipid dependency emerges only after progression to immortalization.

Metabolomic profiling further demonstrated both shared and EBV-augmented responses to hypoxia (Fig 7I). Consistent with HIF1α activation, both EBV and CD40L + IL4 stimulation increased glycolytic PPP intermediates while reducing TCA cycle metabolites. Integrated transcript–metabolite analysis revealed coordinated regulation across major biosynthetic networks (Fig 7I). Upregulation of *SLC7A11* (cystine/glutamate antiporter) corresponded to changes in glutathione homeostasis, indicating that EBV enhances glutathione-based antioxidant capacity but depletes this pool under hypoxic stress. We also observed accumulation of N-carbamoyl-L-aspartate and dihydroorotate, consistent with impaired activity of dihydroorotate dehydrogenase (DHODH), whose function depends on electron transfer through the mitochondrial respiratory chain [80]. In parallel, upregulation of *UPP1*, a key enzyme in pyrimidine salvage, accompanied the buildup of these *de novo* pyrimidine intermediates, suggesting that EBV-infected B-cells may compensate for hypoxia-induced DHODH suppression by reinforcing nucleotide salvage pathways. Together, these metabolite patterns underscore the coupling between EBV-driven transcriptional reprogramming and selective metabolic flux adjustments that recalibrate nucleotide, redox, and energy homeostasis. Moreover, metabolite shifts in creatine and phosphocreatine were unique to EBV-infected cells, indicating virus-specific amplification of energy-buffering capacity.

Together, these data demonstrate that EBV enforces a distinct hypoxia-responsive metabolic state that integrates transcriptional activation of nutrient transporters, biosynthetic enzymes, and antioxidant regulators with coordinated remodeling of lipids and metabolites. This coupled transcriptome–metabolome network enables newly infected B-cells to reprogram energy, redox, and nucleotide metabolism in a manner not fully reproduced by CD40L+IL4 stimulation, establishing a metabolic architecture that likely facilitates adaptation to hypoxic microenvironments and progression toward immortalization.

## Discussion

**Fig 8:**
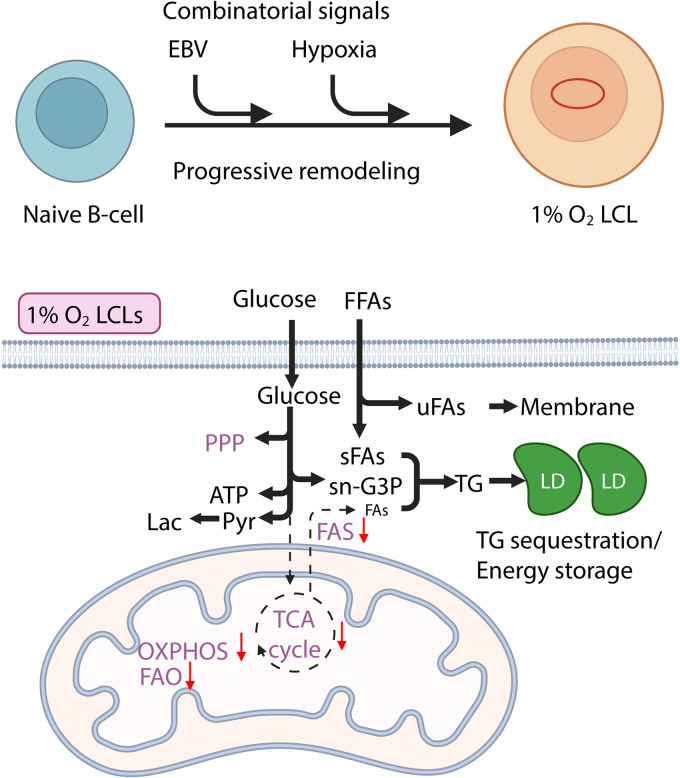
Schematic of metabolic adaptations under hypoxia. FFA, free fatty acid; uFA, unsaturated fatty acid; sFA, saturated fatty acid, Lac, lactate; Pyr, pyruvate; sn-G3P, sn-glyceral-3-phosphate; LD, lipid droplet. This figure was created using biorender.

Our new *ex vivo* transformation model under GC-like hypoxia uncovers a distinct metabolic program that diverges from conventional transformation models and provides a physiologically relevant framework to study EBV-specific metabolic rewiring (Fig 8). From the point of *de novo* infection, EBV and hypoxia cooperatively rewire B-cell metabolism, driving a progressive adaptation that culminates in a hypoxia-specific external lipid addiction phenotype unique to EBV-transformed B-cells.

Beyond the well-characterized metabolic shift toward glycolysis, a critical distinction between EBV transformation at 21% O₂ and 1% O₂ lies in lipid metabolism. Under normoxic conditions, LCLs activate FAS through EBNA2, MYC, and SREBP-driven programs [18]. By contrast, 1% O₂ LCLs exhibit striking suppression of FAS. Mechanistically, this may represent a protective adaptation to avoid lipotoxic stress: saturated fatty acids, such as palmitic acid, are highly toxic in LCLs, and continuous *de novo* synthesis under hypoxia would exacerbate this burden. Consistent with this idea, hypoxic cells redirect metabolism toward external unsaturated fatty acids, such as oleic and linoleic acids, which support proliferation and membrane biogenesis without inducing toxicity. At the same time, they engaged a TG-storage program, marked by elevated TG accumulation and lipid droplet formation, to buffer residual saturated fatty acids. This program was accompanied by strong EBV/hypoxia-specific induction of PLIN2, a lipid droplet–associated structural protein that stabilizes TG stores and limits lipolytic release, thereby preventing lipotoxic stress [81]. Additionally, lipid sequestration has been regulated in part by other HIF-1α-responsive genes, including *HILPDA*, which we found upregulated 13.2-fold in 1% O_2_ LCLs. HILPDA enhances DGAT activity and inhibits ATGL, stabilizing TG stores and mitigating lipotoxicity [82–84]. Notably, this adaptation was not readily observed in CD40L+IL-4–stimulated B-cells under hypoxia in newly infected B-cells, suggesting that EBV’s oncogenic program actively enforces this dependency.

Our findings extend and contextualize prior work on EBV-driven lipid remodeling. Recent studies demonstrated that EBV activates SREBP-dependent mevalonate and fatty acid synthesis pathways early after infection, and that LMP1 alone is sufficient to drive SREBP1/FASN activity and lipid droplet biogenesis, positioning lipogenesis as a core latency output [18,85]. Under normoxia, EBV-driven FASN and desaturation programs (e.g., SCD1/FADS2) are strongly pro-proliferative, an axis recently underscored by Luftig’s group, who identified fatty acid desaturases as key metabolic nodes linking central carbon flux to EBV-transformed B-cell growth [74]. By contrast, our data reveal that physiological hypoxia fundamentally reprograms this canonical circuit: despite intact EBV oncogenic signaling, FAS is curtailed, exogenous unsaturated fatty acids become essential, and protective TG storage is engaged. This framework reconciles prior normoxic models with the germinal center niche. Preservation of mitochondrial function and TG reserves may also enable rapid FAO upon re-oxygenation, potentially explaining why 1% O₂ LCLs proliferate even more rapidly once returned to 21% O₂. Such metabolic flexibility may provide a survival advantage, contributing to the aggressiveness of GC-derived lymphomas transitioning from hypoxic to oxygen-rich environments.

We observed that hypoxia induces profound alterations in redox metabolism. Under hypoxic conditions, the upregulation of the PPP supports NADPH production, enhancing the ability of transformed cells to mitigate excessive ROS. However, EBV infection triggers substantial ROS production under conventional B-cell transformation models, which plays a pivotal role in causing DNA damage necessary for cell transformation and subsequent immortalization into LCLs [86]. The critical involvement of ROS in EBV-mediated B-cell immortalization is underscored by the observation that transformation is significantly impaired in the presence of ROS scavengers [86]. We speculate that physiologically relevant hypoxia may further modulate ROS dynamics during EBV-mediated transformation. This raises the possibility that hypoxia could predispose transformed cells to genomic instability, particularly in long-term residence within hypoxic microenvironments such as GCs. Such instability could facilitate the acquisition of oncogenic mutations, bridging the gap between EBV-mediated transformation and tumorigenesis, ultimately driving the progression from benign hyperproliferation to malignant growth. This hypothesis aligns with observations in EBV-associated malignancies, many of which originate in germinal center B-cells within the hypoxic microenvironment of secondary lymphoid tissues [24]. Hypoxia-induced genomic instability, coupled with the pro-survival and growth-promoting effects of EBV oncoproteins such as LMP1, could create a fertile ground for the emergence of oncogenic mutations. Future investigations are needed, particularly given the well-documented interplay between elevated ROS levels and impaired DNA repair mechanisms under hypoxic conditions in cancer contexts [87].

Despite hypoxia’s established effects on chromatin accessibility, we did not observe significant alterations in EBV-driven SEs at key loci such as MYC and IRF4. This stability suggests that hypoxia-adaptive pathways are layered onto a robust chromatin framework established by viral oncogenes. However, the mechanisms through which the EBV oncogenic program modulates hypoxia adaptation remain to be fully elucidated. Hypoxia has been shown to reshape the DNA methylome by limiting oxygen-dependent TET dioxygenase activity, leading to locus-specific hypermethylation and altered gene expression in diverse cellular contexts. Interestingly, TET2 has been identified as a key factor that demethylates the EBV latent genome, and its expression is restricted to GC B-cells [88,89]. Although we did not directly profile cytosine methylation in this study, prior reports indicate that EBV infection itself engages host methylation machinery, for example through induction of DNMT1 and DNMT3B, which can enforce hypermethylation at select promoters [90]. It is therefore plausible that EBV-infected B-cells under hypoxia experience additional methylation changes, with hypoxia and EBV synergistically shaping the epigenetic landscape. Despite this, we observed that SEs at key EBV-controlled loci (e.g., *MYC*, *IRF4*) remain stable under hypoxia. We hypothesize that this stability reflects not only viral oncogene–anchored enhancer scaffolds (e.g., EBNA2–RBPJ and EBNA-LP–mediated 3D looping), but also contributions from host chromatin remodelers (e.g., BRD4, CHD4/NuRD), DNA methylation writers and erasers (DNMTs, TET enzymes), and hypoxia-responsive transcription factors (HIF-1α, HIF-2α) that may cooperate with EBV oncoproteins such as EBNA2. Together, these layers likely establish a resilient enhancer framework that buffers SE integrity despite fluctuations in oxygen tension. Future studies will be required to define how DNA methylation and chromatin remodeling jointly regulate SE stability in EBV-transformed B-cells.

EBV intrinsically drives metabolic reprogramming by leveraging HIF-1α signaling, with its effects evident even under normoxic conditions, where LMP-driven HIF-1α activation and EBNA3A/LP-mediated HIF-1α stabilization contribute to glycolytic gene expression [91–93]. Under hypoxia, however, HIF-1α activation is markedly amplified, synergizing with the EBV oncogenic program to confer metabolic plasticity to LCLs. This heightened HIF-1α activity under low oxygen not only enhances glycolytic metabolism but also drives the expression of angiogenic and extracellular matrix remodeling genes, such as *VEGFA* and *P4HA1*, equipping cells with the ability to thrive in and adapt to hypoxic microenvironments [94]. The interplay between hypoxia-induced HIF-1α activation and EBV-driven pathways, including NF-κB and PI3K/AKT, fortifies pro-survival and growth-promoting mechanisms, underscoring the critical role of hypoxia in shaping the metabolic and oncogenic landscape of EBV-transformed cells.

In conclusion, hypoxia orchestrates a distinct transformation program in EBV-infected B-cells, marked by profound metabolic reprogramming to prioritize glycolysis, lipid remodeling, and redox balance, promoting survival, proliferation, and potentially tumorigenesis. These findings provide a framework for exploring the intersection of viral oncogenesis, hypoxic adaptation, and tumor progression, offering new insights into the vulnerabilities of EBV-associated malignancies. For example, inhibitors of DGAT or lipid uptake pathways may selectively impair hypoxic-transformed cells in the GCs.

## Materials and methods

### Ethics statement

De-identified human whole blood samples were obtained from Research Blood Components, LLC (Watertown, MA, USA) with approval from IRB: 120160613. Studies on primary human blood cells were approved by the Tufts University Institutional Review Board (Tufts IRB: STUDY00004385).

### Human primary B-cells isolation

Human blood samples were prescreened and confirmed negative for common human pathogens. As the blood samples were de-identified, donor gender was unknown. Primary human B-cells were isolated by negative selection using RosetteSep and EasySep Human B-Cell Enrichment kits (Stem Cell Technologies) following the manufacturers’ protocols. B-cell purity was confirmed by CD19 plasma membrane expression via flow cytometry. Cells were cultured in RPMI 1640 medium (Gibco) with 10% fetal bovine serum (FBS, F31016-500, SeraPrime). Human primary B-cells were stimulated by mitogens including the combination of 1µg/mL anti-human IgM IgG (I0759, Sigma-Aldrich) and 0.5 µM CpG (T*C*G*T*C*G*T*T*T*T*G*T*C*G*T*T*T*T*G*T*C*G*T*T, IDT) or a combination of 50ng/ml CD40L (ALX-522-015-6010, Enzo Life Sciences) and 20ng/ml IL4 (204-IL-050/CF, R&D systems) as described in our previous study [95].

### EBV production and concentration

The EBV B95-8 strain was generated from B95-8 cells engineered for inducible ZTA expression (a generous gift from Dr. Ben Gewurz). The activation of EBV lytic cycle was achieved by treating the cells with 1 μM of 4-hydroxytamoxifen (4HT, Sigma-Aldrich) for 24 hours. Subsequently, the 4HT was removed, and the cells were cultured in RPMI medium supplemented with 10% FBS, devoid of 4HT, for an additional 96 hours. The viral supernatants obtained were then cleared of producer cells by passing through a 0.45 μm filter. The supernatant was transferred to an ultracentrifuge tube (326823, Beckman Coulter) and centrifuged at 25,000 rpm for 2 h at 4°C in an ultracentrifuge (OPTIMA XPN-100, Beckman Coulter). The viral pellet was resuspended and aliquoted in PBS with 2% FBS, stored at −80°C until infection. The genomic DNA of virus was quantified by PCR targeting the BALF5 gene from the extracted viral genome as described [96]. This quantification was used to standardize the virus amounts for cell infection experiments.

### EBV hypoxic transformation model

Purified naïve B-cells were incubated with B95-8 EBV at a multiply of infection (MOI) of 0.1 for 1 hour at room temperature to facilitate viral entry. Following infection, cells were washed with serum free RPMI-1640 medium and resuspended in complete culture medium (RPMI-1640 supplemented with 10% FBS, 2 mM L-glutamine, 100 U/mL penicillin, and 100 μg/mL streptomycin). To assess the impact of oxygen levels on EBV transformation, infected B-cells were cultured under two distinct oxygen conditions: 1) normoxia (21% O₂): cells were maintained in a standard tissue culture incubator (Thermo Fisher Scientific) at 37°C with 5% CO₂. The oxygen level in the incubator was confirmed to be 21%, consistent with atmospheric oxygen at sea level (Boston). 2) hypoxia (1% O₂): cells were placed in a hypoxic incubator (Thermo Fisher Scientific) set to 1% O₂, 5% CO₂, and 94% N₂ at 37°C. Cells were maintained for up to 28 days, during which they proliferated and transformed into lymphoblastoid cell lines (LCLs). Newly infected cells were kept in either 1% or 21% O₂ incubator for the first 7 days without splitting. Established LCLs were subsequently passaged every 3 days by replacing two-thirds of the culture medium with fresh complete RPMI-1640 to sustain growth. All tissue culture handling was done outside of hypoxia incubators (at environmental oxygen tensions of 21% O_2_) as described previously [97].

### Transformation unit assay

Freshly isolated primary human B-cells, purified as outlined above by negative selection, were seeded into two 96-well plates at a density of 20,000 cells/mL in 100 μL per well. The stock of B95.8 EBV was serially diluted tenfold to generate an eight-point dilution series. 100 μL of the virus dilution was added to each well. Plates were placed in either the 21% or 1% O_2_ incubator. Media were refreshed every 3–4 days by carefully aspirating 100 μL of spent media and replenishing it with fresh media. At four weeks post-infection, the proportion of wells with B-cell outgrowth was plotted against the dilution of virus supernatant used per well, as previously described [98]. One transforming unit per well was defined as the amount of virus required to attain B-cell outgrowth in 62.5% of wells.

### Cell viability and growth analysis

#### 1. Cell viability and growth analysis.

Cell viability was assessed using the Countess 3 Automatic Cell Counter (Thermo Fisher Scientific) with Trypan Blue staining (15250061, Thermo Fisher Scientific) to distinguish live from dead cells. For growth curve analysis, live cell counts were recorded at each time point.

To evaluate oxygen-dependent growth dynamics, LCLs from each donor were split into two flasks and cultured in RPMI-1640 medium supplemented with 10% FBS under either 1% or 21% O₂ in dedicated hypoxic or normoxic incubators, respectively.

#### 2. Lipid supplementation studies.

To assess lipid dependency, charcoal-stripped fetal bovine serum (cFBS; A3382101, Gibco) was used to reduce external fatty acid levels. While not completely delipidated, cFBS contains significantly lower levels of free fatty acids and lipophilic molecules compared to regular FBS, allowing controlled lipid supplementation experiments. LCLs cultured under 1% or 21% O₂ were washed twice with DPBS and maintained in RPMI-1640 medium containing 10% cFBS supplemented with either 1 mg/mL lipid-rich BSA (11020039, AlbuMAX I, Gibco) or 1 mg/mL lipid-free BSA (A8806-1G, Sigma-Aldrich).

To define specific lipid requirements under hypoxia, 1% O₂ LCLs were cultured in RPMI-1640 + 10% cFBS supplemented with: (i) 1 mg/mL lipid-rich BSA, (ii) 1 mg/mL lipid-free BSA, (iii) 1 mg/mL oleic/linoleic acid–conjugated BSA (L9655, Sigma-Aldrich), or (iv) 0.36 mg/mL palmitic acid–conjugated BSA (29558, Cayman).

#### 3. Mevalonate pathway inhibition.

To probe the role of the mevalonate pathway, LCLs under both oxygen conditions were treated with 2 µM simvastatin (S1792, Selleckchem) or DMSO control. For rescue experiments, 1% O₂ LCLs were additionally supplemented with 2 µM geranylgeranyl pyrophosphate (GGPP; G6025, Sigma-Aldrich) to assess the requirement for protein prenylation. At the start point, cells were seeded at 3 × 10^5^/mL. Additives were refreshed upon cell splitting.

#### 4. Inhibiting TG biosynthesis.

To assess the role of triglyceride biosynthesis and lipid droplet formation in the survival of LCLs under 1% O₂, cells cultured under both normoxic and hypoxic conditions were treated with 10 µM A922500 (HY-10038, MedChemExpress) or DMSO as a control. At the start point, cells were seeded at 3 × 10^5^/mL. Treatments were refreshed with 10 µM A922500 at 24 and 48 hours post the initial treatment. At 72 hours, cells were counted and harvested for Caspase-3/7 activity analysis.

#### 5. Inhibiting super-enhancers using THZ1.

21% and 1% O₂ LCLs were treated with 100 nM THZ1 (HY-80013, MedChemExpress) or DMSO for 4 days under respective oxygen conditions. At the start point, cells were seeded at 3 × 10^5^/mL. Cell viability was assessed using trypan blue staining followed by automatic cell counting using a Countess 3 cell counter.

#### 6. 6-Aminonicotinamide treatment.

1% or 21% O_2_ LCLs were treated with DMSO or 100 µM 6-Aminonicotinamide, a G6PD inhibitor, (6-AN, No.S9783, Selleckchem) for 48 hours. At the start point, cells were seeded at 3 × 10^5^/mL. Cell viability was assessed using trypan blue staining followed by automatic cell counting using a Countess 3 cell counter.

#### 7. Triacsin C treatment.

To determine the IC50 of Triacsin C (BML-EI218, Enzo) in 21% and 1% O₂ LCLs, cells were seeded into 96-well plates and treated with a range of Triacsin C concentrations (0.001 µM to 100 µM) alongside DMSO-only controls. Plates were incubated at 37°C under their respective oxygen conditions for 72 hours, after which cell viability was assessed using trypan blue staining followed by automatic cell counting using a Countess 3 cell counter. Viability was normalized to the DMSO control, and data were plotted as percentage of live cells versus the log₁₀ of Triacsin C concentration. IC50 values were calculated by fitting a sigmoidal dose-response curve using nonlinear regression.

For all the growth curve analysis, to avoid overconfluency and ensure accurate growth analysis, cultures were regularly split, and total live cell numbers were adjusted based on dilution factors at each passage. Unless otherwise noted, LCLs were consistently maintained in incubators matching their designated oxygen conditions (1% or 21% O₂).

### Chromatin immunoprecipitation (ChIP)

Histone H3K27ac and H3K4me3 ChIP-seq in 1% O₂ and 21% O₂ LCLs was performed using the iDeal ChIP-seq kit for Histones (C01010059, HILOGIC Diagenode) following the manufacturer’s protocol, with ChIP-grade antibodies listed in S1 Table. DNA libraries were prepared using the NEBNext Ultra II DNA Library Prep Kit for Illumina (E7645S, NEB) and sequenced at the Tufts Genomics Core. Read quality was assessed using FastQC to ensure no biases such as GC skew or PCR artifacts. ChIP-seq reads were aligned to the human genome (hg19) using default settings using Bowtie2, except -k was set to 1, with a mappability rate of 94–98% [99]. Peaks were called using MACS v2.1.074 with an FDR threshold of ≤ 0.99 [100], followed by IDR analysis (v2.0.3) with an IDR threshold of ≤ 0.02, as recommended by the ENCODE consortium to ensure peak reproducibility [101]. Peaks in blacklist regions were excluded from downstream analysis. Super-enhancers (SEs) were identified using HOMER under default settings, and ChIP-seq heatmaps were generated using deepTools v3.5.6 [102].

### RNAseq analysis

Total RNA was isolated by the RNeasy Mini kit (Qiagen), following the manufacturer’s manual. An in-column DNA digestion step was included to remove the residual genomic DNA contamination. To construct indexed libraries, 1 µg of total RNA was used for polyA mRNA-selection, using the NEBNext Poly(A) mRNA Magnetic Isolation Module (New England Biolabs), followed by library construction via the NEBNext Ultra RNA Library Prep Kit (New England Biolabs). Each experimental treatment was performed in triplicate. Libraries were multi-indexed, pooled and sequenced on an Illumina NextSeq 500 sequencer using single-end 75 bp reads (Illunima) at the Dana Farber Molecular Biology core. Adaptor-trimmed Illumina reads for each individual library were mapped back to the human GRCh37.83 transcriptome assembly using STAR2.5.2b [103]. Feature Counts was used to estimate the number of reads mapped to each contig [104]. Only transcripts with at least 5 cumulative mapping counts were used in this analysis. DESeq2 was used to evaluate differential expression (DE) [105]. DESeq2 normalizes read counts by estimating size factors that account for differences in sequencing depth and RNA composition across samples, scaling each library relative to the median of the geometric means of counts for all genes. It models normalized counts with a negative binomial distribution to account for biological and technical variability and applies empirical shrinkage to improve dispersion and fold-change estimates. Each DE analysis used pairwise comparison between the experimental and control groups. Differentially expressed genes were identified and a *P*-values < 0.05 and absolute fold change > 2 cutoff was used. For metabolic DE gene analysis, the differentially expressed genes (DEGs) were filtered with a curated metabolic gene list [56]. The total DEGs or metabolic DEGs were subjected to Enrichr analysis which was employed to perform gene list-based gene set enrichment analysis on the selected gene subset. The algorithm used to calculate combined scores was described previously [106]. P value and log_2_ fold change were generated with DESeq2 under default settings with Wald test and normal shrinkage, respectively. Top 5 Enrichr terms that passed the adjusted p-value cutoff were visualized using Graphpad Prism 7. Volcano plots were built with Graphpad Prism7. To concentrate on differentially regulated metabolic pathways, DEGs enriched in 1% O_2_ LCLs were filtered with a curated metabolic gene list [56] and subjected to the STRING protein-protein interaction networks and functional enrichment analysis [107].

### Lipidomic profiling analysis

Lipid profiling was performed as described previously [108,109]. Briefly, 1% and 21% O_2_ LCLs were counted and pelleted at 1,200 rpm for 5 minutes at 4 °C with an equal number of cells in each sample. They were then resuspended in 200 μL of HPLC-grade water (270733, Sigma-Aldrich) and mixed vigorously with 2.5 mL of HPLC-grade methanol (A456, Fisher Scientific) in glass tubes. Following this, 5 mL of methyl tert-butyl ether (MTBE, 1634-04-4, Supelco) was added, and the samples were agitated for 1 hour at room temperature. To separate phases, 1.5 mL of water was added, and after vigorous vortexing, the samples were centrifuged at 1000 x g for 10 minutes at room temperature. The upper phase was then dried a speed vacuum concentrator (Savant SPD 1010, Thermo Fisher Scientific) for 4 h at RT and stored at −80 °C.

For analysis, samples were reconstituted in 35 μL of a 1:1 mixture of LCMS-grade isopropanol and methanol, and subjected to liquid chromatography-mass spectrometry (LC-MS) as previously outlined, employing a high-resolution hybrid QExactive HF Orbitrap mass spectrometer (Thermo Fisher Scientific) set to data-dependent acquisition mode (Top 8) with the capability of switching between positive and negative ion polarities. Lipid species identification and quantification were performed using the LipidSearch 4.1.30 software (Thermo Fisher Scientific), leveraging an internal database comprising ≥20 major lipid classes and ≥80 subclasses. For verifying signal linearity, a pooled sample was created by combining 5 μL from each sample, which was then diluted with a 1:1 mixture of isopropanol and methanol to generate dilutions of 0.3x and 0.1x, alongside a blank. These dilutions underwent analysis, and for each lipid species within this series, the Pearson correlation coefficient between ion count and sample concentration was computed. Only lipids exhibiting a correlation coefficient (r) greater than 0.9 were retained for final analysis. The abundance of individual lipid species was normalized against the total ion count of the sample. Using R, lipids were categorized by class, and the total ion intensity for each lipid class in each sample was calculated.

### Intracellular metabolite profiling

The intracellular metabolites profiling was performed as described [110]. 1% and 21% O_2_ LCLs were washed 3 times with pre-chilled PBS and counted. The cell pellet was fully resuspended with 100uL PBS by vortex, the metabolism was quenched by adding 3.3 mL of dry ice-cold 80% aqueous methanol (A456, Fisher Scientific), and kept at -80°C overnight. The lysate was centrifuged at 21,000 g for 15 min at 4°C. The supernatants were obtained and dried by a speed vacuum concentrator (Savant SPD 1010, Thermo Fisher Scientific) for 4 hours at RT. Samples were re-suspended using 20 uL HPLC grade water for mass spectrometry. 5–7 μL were injected and analyzed using a hybrid 6500 QTRAP triple quadrupole mass spectrometer (AB/SCIEX) coupled to a Prominence UFLC HPLC system (Shimadzu) via selected reaction monitoring (SRM) of a total of 300 endogenous water soluble metabolites for steady-state analyses of samples. Some metabolites were targeted in both positive and negative ion mode for a total of 311 SRM transitions using positive/negative ion polarity switching. ESI voltage was + 4950V in positive ion mode and –4500V in negative ion mode. The dwell time was 3 ms per SRM transition and the total cycle time was 1.55 seconds. Approximately 9–12 data points were acquired per detected metabolite. Samples were delivered to the mass spectrometer via hydrophilic interaction chromatography (HILIC) using a 4.6 mm i.d x 10 cm Amide XBridge column (Waters) at 400 μL/min. Gradients were run starting from 85% buffer B (HPLC grade acetonitrile) to 42% B from 0-5 minutes; 42% B to 0% B from 5-16 minutes; 0% B was held from 16-24 minutes; 0% B to 85% B from 24-25 minutes; 85% B was held for 7 minutes to re-equilibrate the column. Buffer A was comprised of 20 mM ammonium hydroxide/20 mM ammonium acetate (pH = 9.0) in 95:5 water:acetonitrile. Peak areas from the total ion current for each metabolite SRM transition were integrated using MultiQuant v3.0.2 software (AB/SCIEX). Metabolites with p-values < 0.05, log2(fold change)>1 or <-1 were used for pathway analysis using MetaboAnalyst 5.0 (https://www.metaboanalyst.ca/MetaboAnalyst/ModuleView.xhtml). Heatmaps were generated by feeding Z-score values of selected metabolites into Morpheus software (https://software.broadinstitute.org/morpheus/).

### Seahorse mitochondrial stress test

The Agilent Seahorse XF Assay was conducted as described preiously [14]. Specifically, the sensor cartridge was first hydrated with water overnight and incubated with XF Calibrant for 1h. Add 12 µL Cell-Tak solution (1.3 mL of 0.1M sodium bicarbonate, 11.2 µL of 0.1M NaOH, 22.4 µL of Cell-Tak solution) to each well of the V7-PS 96-well cell culture plate. The Cell-Tak solution was washed with sterile water twice and 0.25 million 1% and 21% O_2_ LCLs (resuspension in 180 µL of RPMI-1640 with 10% FBS and 5 mM pyruvate) were seed on a Seahorse plate. Then the cells were placed in a non-CO_2_ 37 °C for 30 minutes. The oxygen consumption rates (OCR) and extracellular acidification rate (ECAR) were simultaneously recorded by a Seahorse XFe96 Analyzer (Agilent). The cells were sequentially probed by 20 µL of 3.5 µM oligomycin A (No.S1478, Selleckchem), 20 µL of 2 µM CCCP (No.S6494, Selleckchem), and 20 µL of 100 nM piericidin A (HY-114936, MedChemExpress). Data was analyzed by Seahorse Wave Desktop Software (Agilent).

### Flow cytometry analysis

The mitochondrial mass was determined by the MitoTracker Green FM (M7514, Thermo Fisher Scientific) following the manual. 1 × 10^6^ of Cells were collected and resuspended in 500 μL cell culture media with 1.5 μL 100 μM of MitoTracker Green. Cells were then incubated in 37°C incubator for 30 min. Then cells were washed once with 1 × PBS and resuspended in PBS buffer with 2% FBS for FACS. For CFSE (C345544, Invitrogen) cell proliferation staining, 10 million of primary B-cells were resuspended in PBS with 0.1% BSA, then the cells were mixed with the same volume of 1μM CFSE for 10 min at 37°C. Cells were then neutralized by prechilled 10% FBS RMPI-1640 for 5 min. After washing the cell with culture media, cells were resuspended and infected with EBV. 1 h after infection, cells were treated with 1% or 21% O_2_ for 5 or 7 days. As controls of uninfected B-cells, CFSE stained primary B-cells were stimulated with a combination of anti-human IgM IgG and CpG or a combination of CD40L and IL4 and cultured in a 1% or 21% O_2_ incubator for 5 days. Flow cytometry was performed on a BD FACS Calibur instrument. Data was analyzed with FlowJo V10. Only live cells were included in the analysis based on FSC/SSC gating to exclude dead cells and debris.

### Caspase activation assay

Caspase 3/7 activity was quantified by Caspase-Glo assays (G8090, Promega) according to manufacturer’s manual and normalized to the cell number of the same sample determined by Trypan Blue staining and cell counting using an automatic cell counter Countess 3 (Thermo Fisher Scientific). All values were quantitated on a Promega GloMax Plate Reader (Promega).

### Western blot analysis

Immunoblot analysis was performed according to the previous methods [98]. Cell lysates were prepared by incubating cells in 1 × Laemmli buffer at 95 °C for 5 min. Lysate Samples were separated by SDS-PAGE electrophoresis, transferred onto the nitrocellulose membranes, blocked with 5% milk in TBST buffer for 1 h, and then probed with relevant primary antibodies at 4°C overnight. Restore Western Blot Stripping Buffer (21063, Thermo Fisher Scientific) was used when necessary. The next day, the membranes were incubated with secondary antibody for 1 h. Blots were then developed by incubation with ECL chemiluminescence (Millipore) and images were captured by Licor Fx system. Bands intensities were measured where indicated by Image Studio Lite Version 5.2. All antibodies used in this study were listed S6 Table.

### Confocal microscopy

1% O_2_ LCLs, 21% O_2_ LCLs treated with DMSO or 10 µM DGAT1 inhibitor, A922500 (HY-10038, MedChemExpress) for 24 hours were supplemented with 10 µM Bodipy FL C12 (D3822, Thermo Fisher Scientific) for 30min. Cells were then washed once with PBS and resuspended in RMPI-1640 with 10% charcoal-stripped FBS for live cell confocal imaging with Zeiss LSM900. To ensure precise timing after Bodipy FL C12 treatment, experiments were conducted one cell line at a time.

### Quantification and statistical analysis

Unless otherwise indicated, all bar graphs and line graphs represent the arithmetic mean of three independent experiments (n = 3), with error bars denoting standard deviations. Data were analyzed using unpaired Student t-test or analysis of variance (ANOVA) with the appropriate post-test using GraphPad Prism7 software. Gene ontology analysis was done with the Enrichr module using the KEGG pathway databases. Default parameters of Enrichr module was used, with the exception that the Enrichment statistic was set as classic. Metabolic pathway analysis was performed using MetaboAnalyst 6.0. Figures were drawn with commercially available GraphPad, Biorender, Microsoft Powerpoint.

### Graphics

Figures were drawn with GraphPad, Biorender, Microsoft Powerpoint, and ggplot2 in R.

## Supporting information

S1 Table Super-enhancer analysis of H3K27ac ChIP from 1% or 21% O₂ LCL derived from donor 1. (XLSX)

S2 Table RNA sequencing analysis of mRNA transcripts from 1% or 21% O₂ LCL derived from donors 1, 2, and 3. (XLSX)

S3 Table LC/MS analysis of intracellular metabolites from 1% or 21% O₂ LCL derived from donor 1. (XLSX)

S4 Table Lipidomic analysis of intracellular lipids from 1% or 21% O₂ LCL derived from donor 1. (XLSX)

S5 Table RNA sequencing, metabolomic, and lipidomic analysis of primary B-cells infected with EBV or stimulated with CD40L + IL-4 and cultured under 1% or 21% O₂ incubator for 5-days. Primary B-cells were isolated from whole blood from donor 4.
(XLSX)

S6 Table Antibodies used in the study. (DOCX)

S1 Fig EBV maintains viral oncogene expression and super-enhancer (SE) establishment regardless of oxygen levels, related to Fig 2. A. Immunoblot analysis for indicated proteins in whole cell lysates (WCL) in EBV newly transformed human primary B-cells, collected at indicated days post-infection in either 1% or 21% O_2_. These represent reproducible experiments from donor 3. B. ChIP-seq heatmap of H3K4me3 and H3K27ac in LCLs under 1% and 21% O₂ LCLs. Heatmaps display histone modification signals across four gene clusters (Cl1–Cl4) centered on transcription start (TSS) and end sites (TES). Color scale represents log2-transformed signal intensity relative to input. Heatmap represents the mean relative peak intensity from n = 2 experiments. C. H3K27ac and H3K4me3 ChIP-seq tracks from 1% O_2_, 21% O_2_, or GM12878 LCL, and GM12878 ChIA-PET Pol ll tracks are shown. The black arrow indicates *IRF4* or *BCL2* loci. Black boxes indicate SEs.
(TIF)

S2 Fig RNA-seq Reveals Hypoxia-Specific Transcriptomic Adaptations in 1% O_2_ LCLs, related to Fig 3. A. RNA-seq validation of 1% O₂ and 21% O₂ LCLs established from three independent donors (n = 1 LCL per donor). The initial RNA-seq analysis in Fig 3 was performed using LCLs derived from donor 1. To validate these findings, RNA-seq was repeated using independently generated 1% or 21% O_2_ LCLs from two additional donors under identical culture conditions. Shown are the fold changes of representative PC1-associated genes originally identified from the donor 1 dataset. B.STRING network analysis [107] of selected DEGs filtered by a curated metabolic gene list [56] upregulated in 1% O_2_ LCLs. Edges represent protein-protein associations. Confidence scores, which are scaled between 0–1, indicate the strength of data support. Confidence score values indicate the estimated likelihood that a given interaction is biologically meaningful, specific and reproducible given the supporting evidence, and are indicated according to the key at the bottom left.
(TIF)

S3 Fig Hypoxia alters redox balance in 1% O_2_ LCLs, related to Fig 4. A. FACS analysis of MitoTracker Green MFI in 1% or 21% O_2_ LCLs. Mean + /- SD values were from n = 3 experiments using LCLs from donor 1 but experiments were repeated with >2 additional donors. P-values were calculated using an unpaired Student’s t-test. B. Bar chart analysis of NADP + ion intensity, NADPH ion intensity, and NADPH/NADP+ ratios in 1% or 21% O₂ LCLs. Mean + /- SD values are from n = 3 metabolomics experiments from donor 1 LCLs. P-values were calculated using an unpaired Student’s t-test. C. Absolute live cell number of 1% or 21% O_2_ LCLs treated with DMSO or 100 µM 6-AN for 48 hours. Cells were seeded at 3 × 10^5^/mL. Mean + /- SD values are from n = 3 experiments from donor 1 LCLs. P-values were determined using two-way ANOVA with Sidak’s multiple comparisons test. D. Bar chart analysis of NAD + ion intensity, NADH ion intensity, and NADH/NAD+ ratios in 1% or 21% O₂ LCLs. Mean + /- SD values are from n = 3 metabolomics experiments from donor 1 LCLs. P-values were determined using an unpaired Student’s t-test. E. Bar chart analysis of DESeq2 normalized RNAseq reads of *LDHA* in 1% or 21% O_2_ LCLs. Mean + /- SD values were from n = 3 RNAseq experiments from donor 1 LCLs. P-values were calculated using an unpaired Student’s t-test.
(TIF)

S4 Fig Hypoxia induces TG biosynthesis and lipid droplet formation in 1% O₂ LCLs, related to Fig 5. A. Bar chart analysis of DESeq2 normalized RNAseq reads of indicated genes in 1% or 21% O_2_ LCLs. Mean + /- SD values were from n = 3 RNAseq experiments from donor 1 LCLs. P-values were calculated using two-way ANOVA with Sidak’s multiple comparisons test. B. Confocal microscopic analysis of 1% O_2_ LCLs treated with DMSO or 10 µM A922500, a DGAT1 inhibitor for 24 hours. Cells were then treated with 10 µM Bodipy FL C12 for 20 min prior to the imaging. Representative of n = 3 experiments using donor 1 LCLs. Scale bar, 10 µm. C. Fold change of live cell number in 1% or 21% O_2_ LCLs treated with DMSO or 10 µM DGAT1 inhibitor (A922500) for 72 hours. Mean + /- SD values were from n = 3 experiments using donor 1 LCLs. P-values were calculated using two-way ANOVA with Holm-Sidak’s multiple comparisons test. D. Caspase 3/7 activity in 1% or 21% O_2_ LCLs treated with DMSO or 10 µM DGAT1 inhibitor (A922500) for 72 hours. Mean + /- SD values were from n = 3 experiments using donor 1 and 2 LCLs. P-values were calculated using two-way ANOVA with Holm-Sidak’s multiple comparisons test.
(TIF)

S5 Fig Extracellular unsaturated fatty acids are essential for the survival of 1% O_2_ LCLs, related to Fig 6. A. Bar chart analysis of DESeq2 normalized RNAseq reads of indicated genes in 1% or 21% O_2_ LCLs. Mean + /- SD values were from n = 3 RNAseq experiments from donor 1 LCLs. P-values were calculated using an unpaired Student’s t-test. B. Growth curve of 1% O₂ LCLs from two additional donors cultured in indicated condition. Abbreviations in the culture condition: 1% O_2_, 1% O_2_ incubator; RPMI, RPMI-1640 media; cFBS, 10% charcoal-stripped FBS; Lipid-rich BSA, 1 mg/mL; Lipid-free BSA, 1mg/mL; Linoleic/oleic acids BSA, 1 mg/mL; Palmitic acid BSA, 0.36 mg/mL. Mean + /- SD values are from n = 3 experiments using donor 2 and 3 LCLs. P-values were calculated using two-way ANOVA with Dunnett’s multiple comparisons test, comparing each group to the 1% O₂ LCL cultured under lipid rich media. C. Palmitic acid dose–response assay in LCLs cultured under 21% O₂ or 1% O₂ conditions. Cells were treated with the indicated concentrations of palmitate–BSA conjugate and maintained under the specified oxygen conditions for 72 hours. Cell viability was assessed by Trypan Blue exclusion using an automated cell counter. Data represent mean ± SD from three independent experiments using LCLs derived from donors 2 and 3. D. Caspase 3/7 activity in 1% O_2_ LCLs cultured in the lipid free media repleted with lipid-rich, lipid-free, oleic/linoleic acid, or palmitic acid BSA as in B. Mean + /- SD values were from n = 3 experiments using donor 2 or 3 LCLs. P-values were calculated using one-way ANOVA with Holm-Sidak’s multiple comparisons test.
(TIF)

S6 Fig IC50 analysis of Triacsin C treatment in 1% O₂ or 21% O₂ LCLs. Data represent mean ± SD from three independent experiments using LCLs derived from donors 1, 2, and 3. The corresponding confidence intervals (CI) are also shown.
(TIF)

S7 Fig Selection gene transcription in 1% or 21% O_2_ LCLs derived form independent donors. RNAseq read count of *CD36*, *SLC27A1 (FATP1), SLC27A4 (FATP4), GPR84*, *GPR120* (*FFAR4*), *GPR183* (*EBI2*) mRNAs in 1% or 21% O_2_ LCLs derived from donor 1, 2, and 3. Data is from n = 1 RNAseq replicate from independent donors.
(TIF)

S8 Fig Mevalonate pathway remains critical for protein prenylation in 1% O_2_ LCLs. A. Bar chart analysis of DESeq2 normalized RNAseq reads of indicated genes in 1% or 21% O_2_ LCLs. Mean + /- SD values were from n = 3 experiments. P-values were calculated using an unpaired Student’s t-test. B. Bar chart analysis of acetoacetyl-CoA and Cholesterol ion intensity in 1% or 21% O_2_ LCLs. Mean + /- SD values were from n = 3 experiments using donor 1 LCLs. P-values were calculated using an unpaired Student’s t-test. C. A schematic representation of mevalonate pathway. The pathway illustrates the conversion of Acetyl-CoA to cholesterol and isoprenoids via HMG-CoA reductase (HMGCR), a key regulatory enzyme inhibited by Simvastatin. Mevalonate serves as a precursor for farnesyl pyrophosphate, which branches into cholesterol biosynthesis (via squalene) and protein prenylation (via geranylgeranyl pyrophosphate, GGPP). LDLR (low-density lipoprotein receptor) regulates extracellular cholesterol uptake. D. Growth curve of 1% or 21% O_2_ LCLs treated with DMSO or 2 µM Simvastatin. Cells were seeded at 3 × 10^5^/mL. Mean + /- SD values were from n = 3 experiments using donor 1 LCLs. P-values were calculated using a two-way ANOVA with Tukey’s multiple comparisons test. E-G. Growth curve of 1% O_2_ LCLs treated with DMSO, 2 µM Simvastatin or 2 µM Simvastatin plus 2 µM GGPP. Cells were seeded at 3 × 10^5^/mL. Mean + /- SEM values were from n = 3 experiments using LCLs derived from donor 1, 2, and 3. P-values were calculated using a two-way ANOVA with Dunnet’s multiple comparisons test, comparing each group to the 1% O₂ LCL cultured under simvastatin.
(TIF)
