# Supplementary material for: Epstein-Barr virus-transformed B-cells from a hypoxia model of the germinal center requires external unsaturated fatty acids
Source: PLoS Pathog. 2025 Nov 11;21(11):e1013694. doi: 10.1371/journal.ppat.1013694 (PMC12626287; doi:10.1371/journal.ppat.1013694)
Supplement: S6 Table — (DOCX) [file ppat.1013694.s006.docx]

**Table S6**

**Antibodies**

| **Antibodies** | **Vendor** | **Identifier** | **RRID** |
| --- | --- | --- | --- |
| Anti-c-MYC (9E10) mouse monoclonal IgG1 | Santa Cruz Biotechnology | Cat# sc-40 | AB_627268 |
| Anti-DDX1 rabbit polyclonal antibody | Bethyl Laboratories | Cat# AB_451046 | AB_451046 |
| Anti-Beta actin rabbit recombinant antibody | Proteintech | Cat# 81115-1-RR | AB_2923704 |
| Anti-EBNA2 CLONE R3 rat monoclonal antibody | MilliporeSigma | Cat# MABE8 | AB_10807963 |
| Anti-IRF4 (E8H3S) rabbit monoclonal antibody | Cell Signaling Technology | Cat# 62834 | AB_2877647 |
| Anti-EBV Latent Membrane Protein 1 antibody mouse monoclonal antibody | MilliporeSigma | Cat# MABF2248 | AB_3662815 |
| Anti-CHOP (D46F1) Rabbit mAb | Cell Signaling Technology | Cat# 5554S | AB_10694399 |
| H3K27ac antibody, ChIP Grade | Diagenode | Cat# C15410196 | AB_2637079 |
| H3K4me3 antibody, ChIP Grade | Diagenode | Cat# C15410003 | AB_2924768 |
| Anti-Rabbit IgG HRP-coupled secondary antibody | Cell Signaling Technology | Cat# 7074S | AB_2099233 |
| Anti-Mouse IgG HRP-coupled secondary antibody | Cell Signaling Technology | Cat# 7076S | AB_330924 |
| Anti-Rat IgG HRP-coupled secondary antibody | Cell Signaling Technology | Cat# 7077S | AB_10694715 |
