# Supplementary figures and images for: Epstein-Barr virus-transformed B-cells from a hypoxia model of the germinal center requires external unsaturated fatty acids
Source: PLoS Pathog. 2025 Nov 11;21(11):e1013694. doi: 10.1371/journal.ppat.1013694 (PMC12626287; doi:10.1371/journal.ppat.1013694)

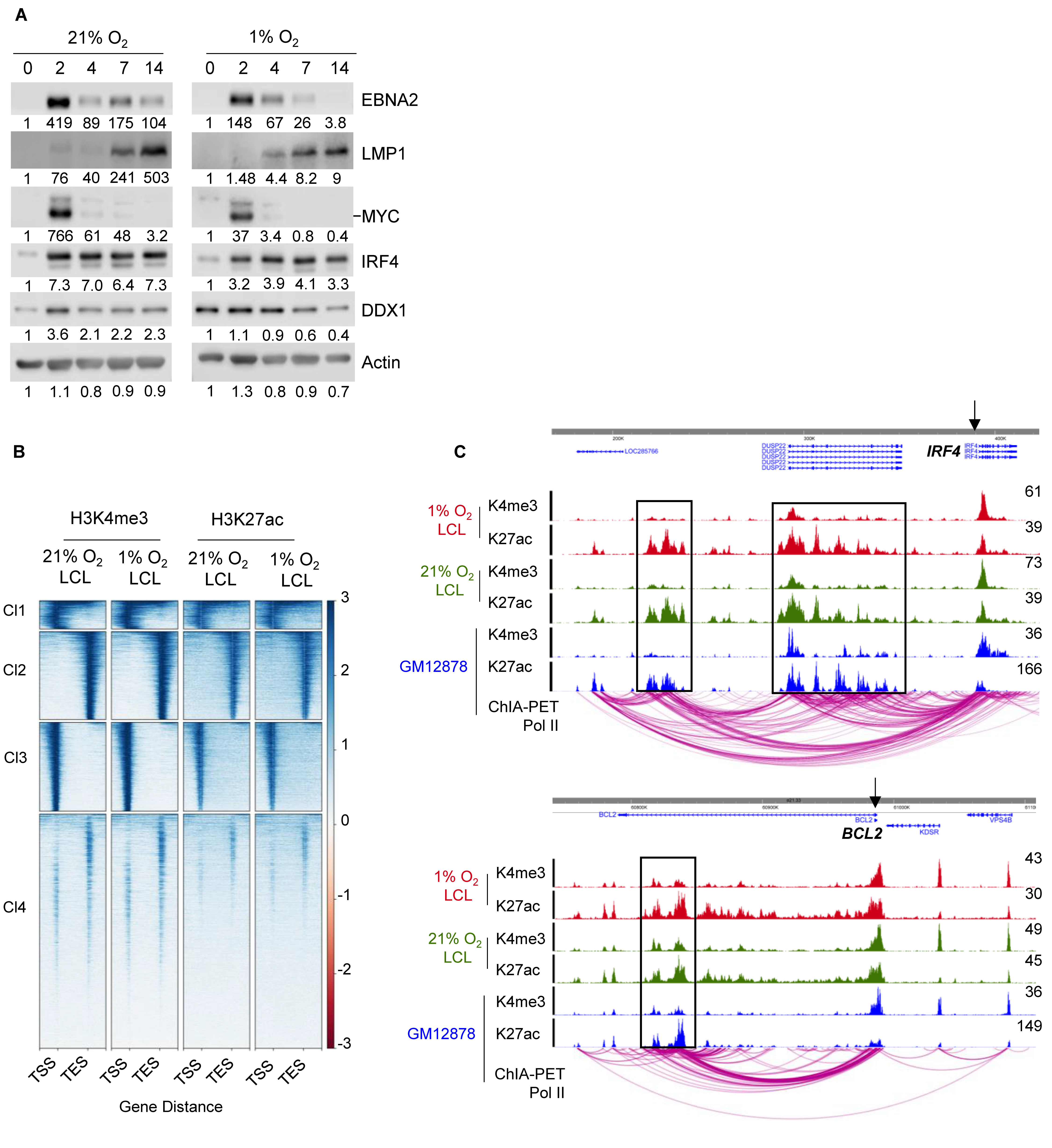

Supplement: S1 Fig — A. Immunoblot analysis for indicated proteins in whole cell lysates (WCL) in EBV newly transformed human primary B-cells, collected at indicated days post-infection in either 1% or 21% O2. These represent reproducible experiments from donor 3. B. ChIP-seq heatmap of H3K4me3 and H3K27ac in LCLs under 1% and 21% O₂ LCLs. Heatmaps display histone modification signals across four gene clusters (Cl1–Cl4) centered on transcription start (TSS) and end sites (TES). Color scale represents log2-transformed signal intensity relative to input. Heatmap represents the mean relative peak intensity from n = 2 experiments. C. H3K27ac and H3K4me3 ChIP-seq tracks from 1% O2, 21% O2, or GM12878 LCL, and GM12878 ChIA-PET Pol ll tracks are shown. The black arrow indicates IRF4 or BCL2 loci. Black boxes indicate SEs. (TIF) [file ppat.1013694.s007.tif]

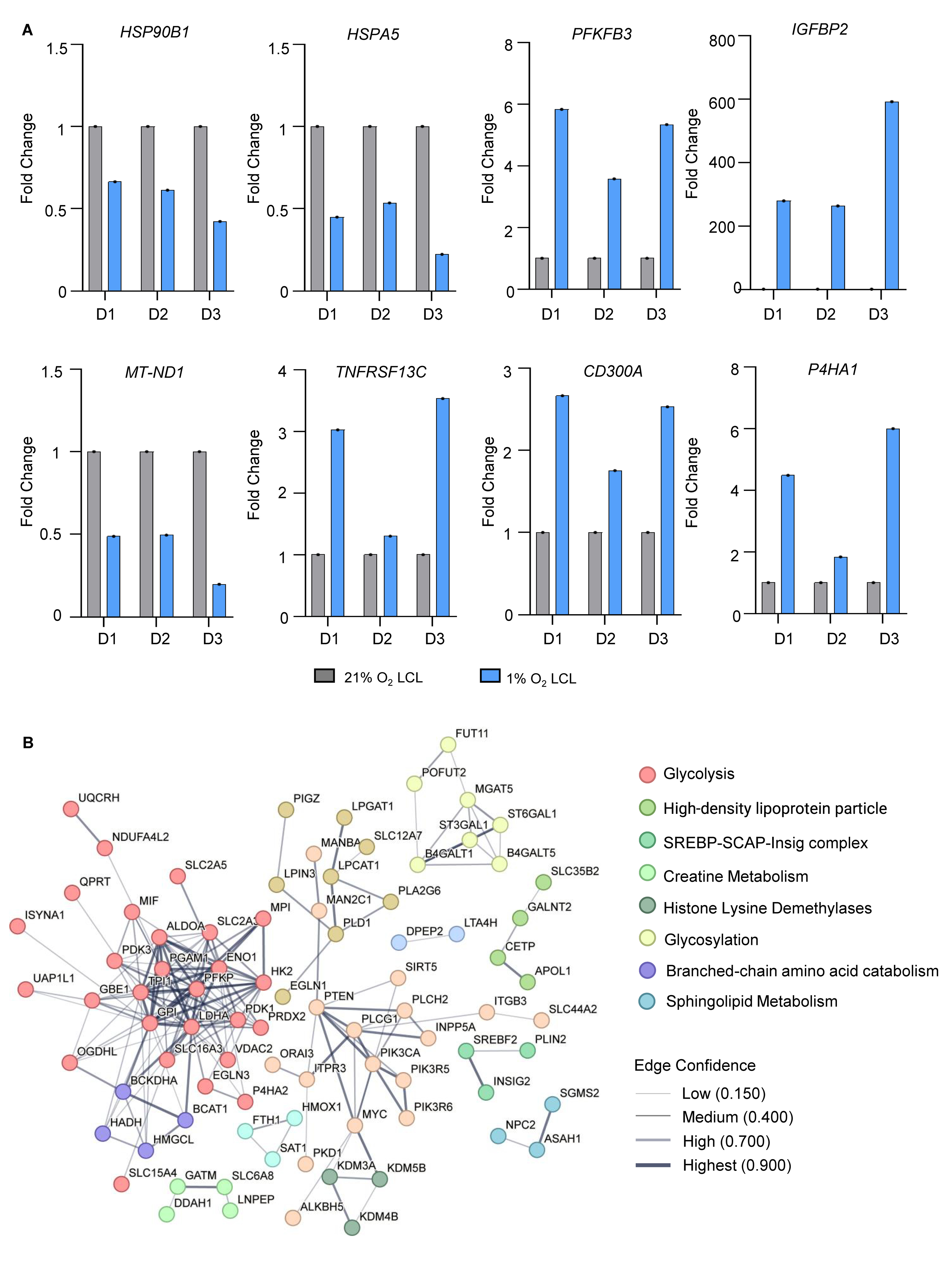

Supplement: S2 Fig — A. RNA-seq validation of 1% O₂ and 21% O₂ LCLs established from three independent donors (n = 1 LCL per donor). The initial RNA-seq analysis in Fig 3 was performed using LCLs derived from donor 1. To validate these findings, RNA-seq was repeated using independently generated 1% or 21% O2 LCLs from two additional donors under identical culture conditions. Shown are the fold changes of representative PC1-associated genes originally identified from the donor 1 dataset. B.STRING network analysis [107] of selected DEGs filtered by a curated metabolic gene list [56] upregulated in 1% O2 LCLs. Edges represent protein-protein associations. Confidence scores, which are scaled between 0–1, indicate the strength of data support. Confidence score values indicate the estimated likelihood that a given interaction is biologically meaningful, specific and reproducible given the supporting evidence, and are indicated according to the key at the bottom left. (TIF) [file ppat.1013694.s008.tif]

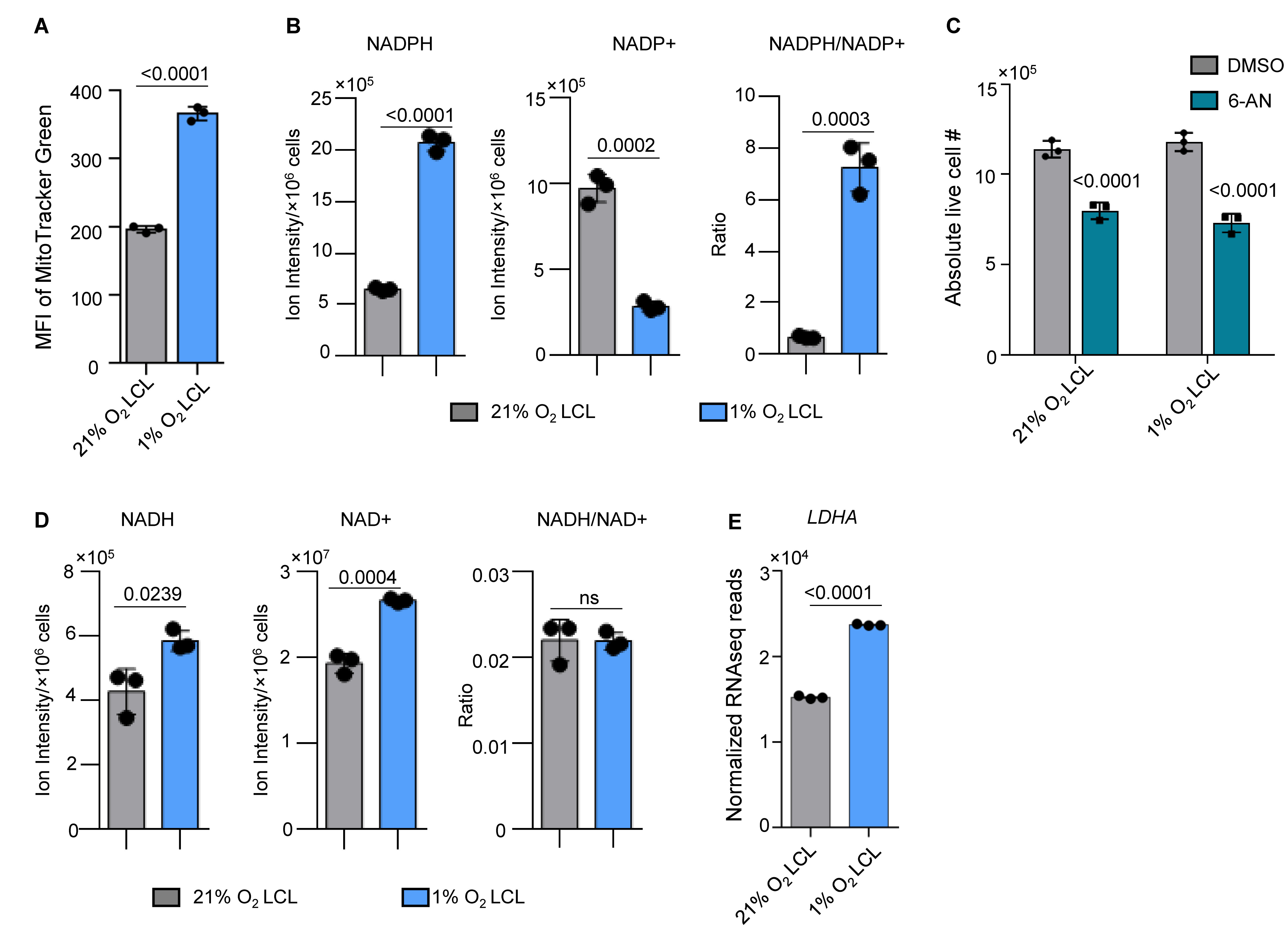

Supplement: S3 Fig — A. FACS analysis of MitoTracker Green MFI in 1% or 21% O2 LCLs. Mean + /- SD values were from n = 3 experiments using LCLs from donor 1 but experiments were repeated with >2 additional donors. P-values were calculated using an unpaired Student’s t-test. B. Bar chart analysis of NADP + ion intensity, NADPH ion intensity, and NADPH/NADP+ ratios in 1% or 21% O₂ LCLs. Mean + /- SD values are from n = 3 metabolomics experiments from donor 1 LCLs. P-values were calculated using an unpaired Student’s t-test. C. Absolute live cell number of 1% or 21% O2 LCLs treated with DMSO or 100 µM 6-AN for 48 hours. Cells were seeded at 3 × 10^5^/mL. Mean + /- SD values are from n = 3 experiments from donor 1 LCLs. P-values were determined using two-way ANOVA with Sidak’s multiple comparisons test. D. Bar chart analysis of NAD + ion intensity, NADH ion intensity, and NADH/NAD+ ratios in 1% or 21% O₂ LCLs. Mean + /- SD values are from n = 3 metabolomics experiments from donor 1 LCLs. P-values were determined using an unpaired Student’s t-test. E. Bar chart analysis of DESeq2 normalized RNAseq reads of LDHA in 1% or 21% O2 LCLs. Mean + /- SD values were from n = 3 RNAseq experiments from donor 1 LCLs. P-values were calculated using an unpaired Student’s t-test. (TIF) [file ppat.1013694.s009.tif]

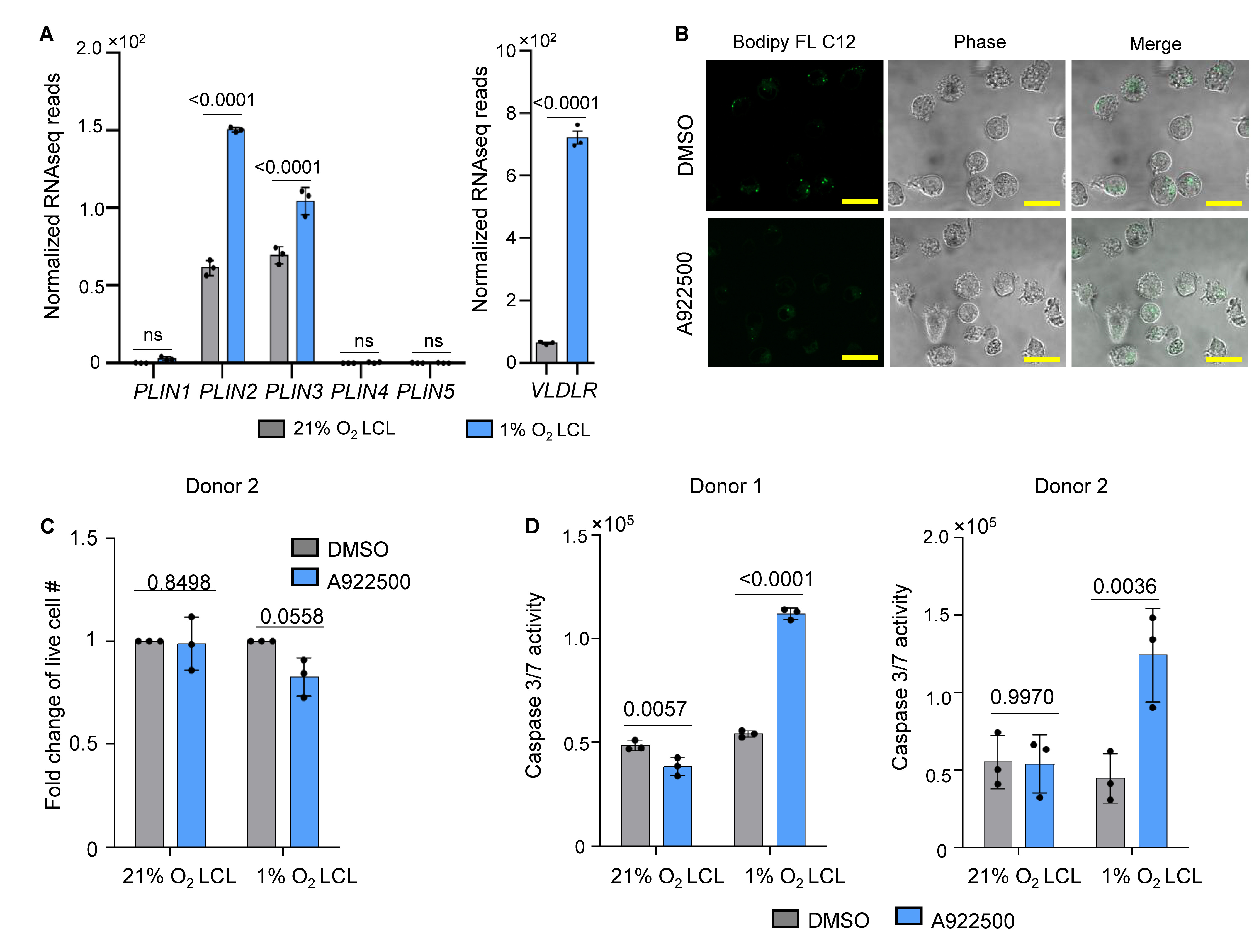

Supplement: S4 Fig — A. Bar chart analysis of DESeq2 normalized RNAseq reads of indicated genes in 1% or 21% O2 LCLs. Mean + /- SD values were from n = 3 RNAseq experiments from donor 1 LCLs. P-values were calculated using two-way ANOVA with Sidak’s multiple comparisons test. B. Confocal microscopic analysis of 1% O2 LCLs treated with DMSO or 10 µM A922500, a DGAT1 inhibitor for 24 hours. Cells were then treated with 10 µM Bodipy FL C12 for 20 min prior to the imaging. Representative of n = 3 experiments using donor 1 LCLs. Scale bar, 10 µm. C. Fold change of live cell number in 1% or 21% O2 LCLs treated with DMSO or 10 µM DGAT1 inhibitor (A922500) for 72 hours. Mean + /- SD values were from n = 3 experiments using donor 1 LCLs. P-values were calculated using two-way ANOVA with Holm-Sidak’s multiple comparisons test. D. Caspase 3/7 activity in 1% or 21% O2 LCLs treated with DMSO or 10 µM DGAT1 inhibitor (A922500) for 72 hours. Mean + /- SD values were from n = 3 experiments using donor 1 and 2 LCLs. P-values were calculated using two-way ANOVA with Holm-Sidak’s multiple comparisons test. (TIF) [file ppat.1013694.s010.tif]

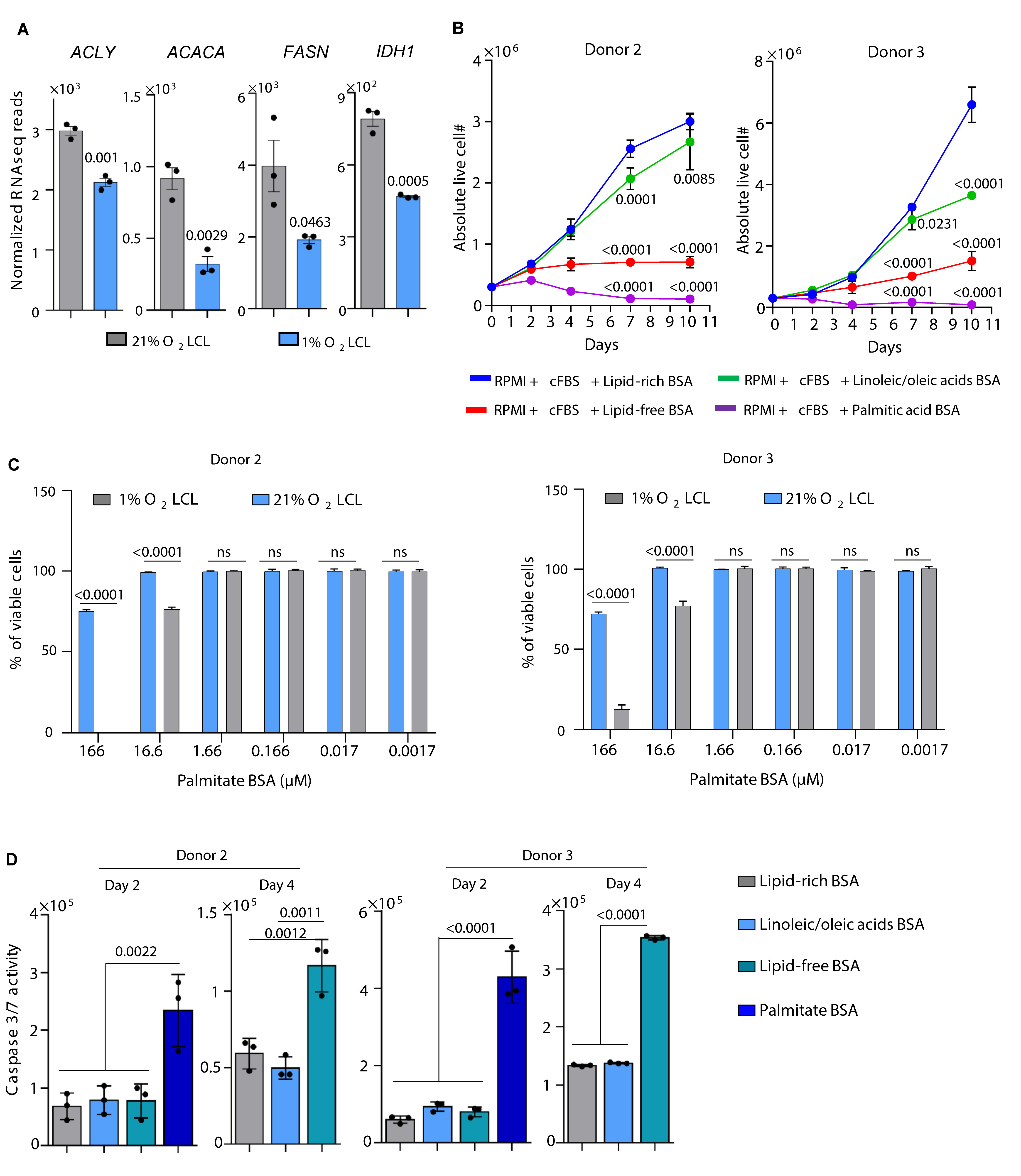

Supplement: S5 Fig — A. Bar chart analysis of DESeq2 normalized RNAseq reads of indicated genes in 1% or 21% O2 LCLs. Mean + /- SD values were from n = 3 RNAseq experiments from donor 1 LCLs. P-values were calculated using an unpaired Student’s t-test. B. Growth curve of 1% O₂ LCLs from two additional donors cultured in indicated condition. Abbreviations in the culture condition: 1% O2, 1% O2 incubator; RPMI, RPMI-1640 media; cFBS, 10% charcoal-stripped FBS; Lipid-rich BSA, 1 mg/mL; Lipid-free BSA, 1mg/mL; Linoleic/oleic acids BSA, 1 mg/mL; Palmitic acid BSA, 0.36 mg/mL. Mean + /- SD values are from n = 3 experiments using donor 2 and 3 LCLs. P-values were calculated using two-way ANOVA with Dunnett’s multiple comparisons test, comparing each group to the 1% O₂ LCL cultured under lipid rich media. C. Palmitic acid dose–response assay in LCLs cultured under 21% O₂ or 1% O₂ conditions. Cells were treated with the indicated concentrations of palmitate–BSA conjugate and maintained under the specified oxygen conditions for 72 hours. Cell viability was assessed by Trypan Blue exclusion using an automated cell counter. Data represent mean ± SD from three independent experiments using LCLs derived from donors 2 and 3. D. Caspase 3/7 activity in 1% O2 LCLs cultured in the lipid free media repleted with lipid-rich, lipid-free, oleic/linoleic acid, or palmitic acid BSA as in B. Mean + /- SD values were from n = 3 experiments using donor 2 or 3 LCLs. P-values were calculated using one-way ANOVA with Holm-Sidak’s multiple comparisons test. (TIF) [file ppat.1013694.s011.tif]

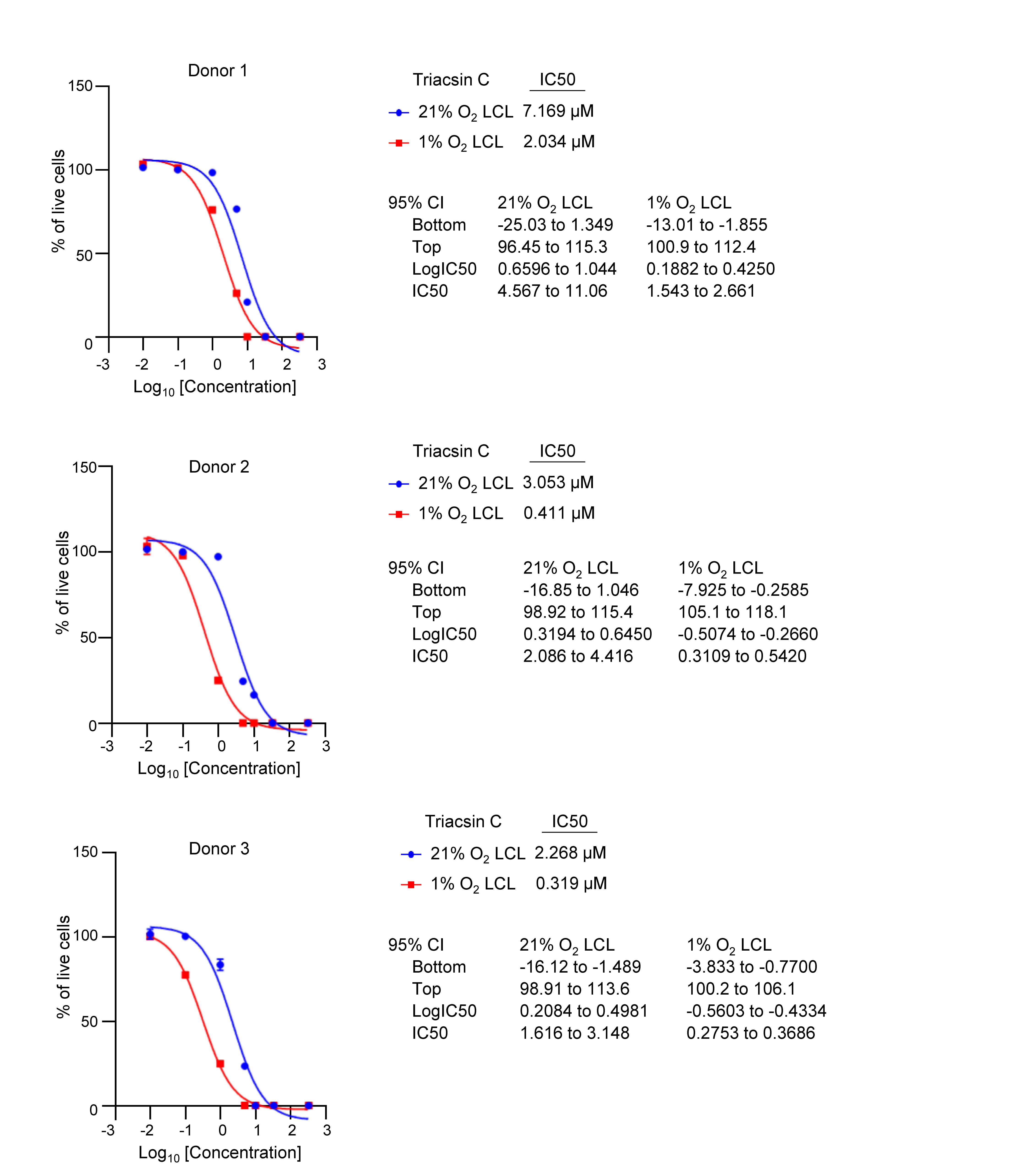

Supplement: S6 Fig — Data represent mean ± SD from three independent experiments using LCLs derived from donors 1, 2, and 3. The corresponding confidence intervals (CI) are also shown. (TIF) [file ppat.1013694.s012.tif]

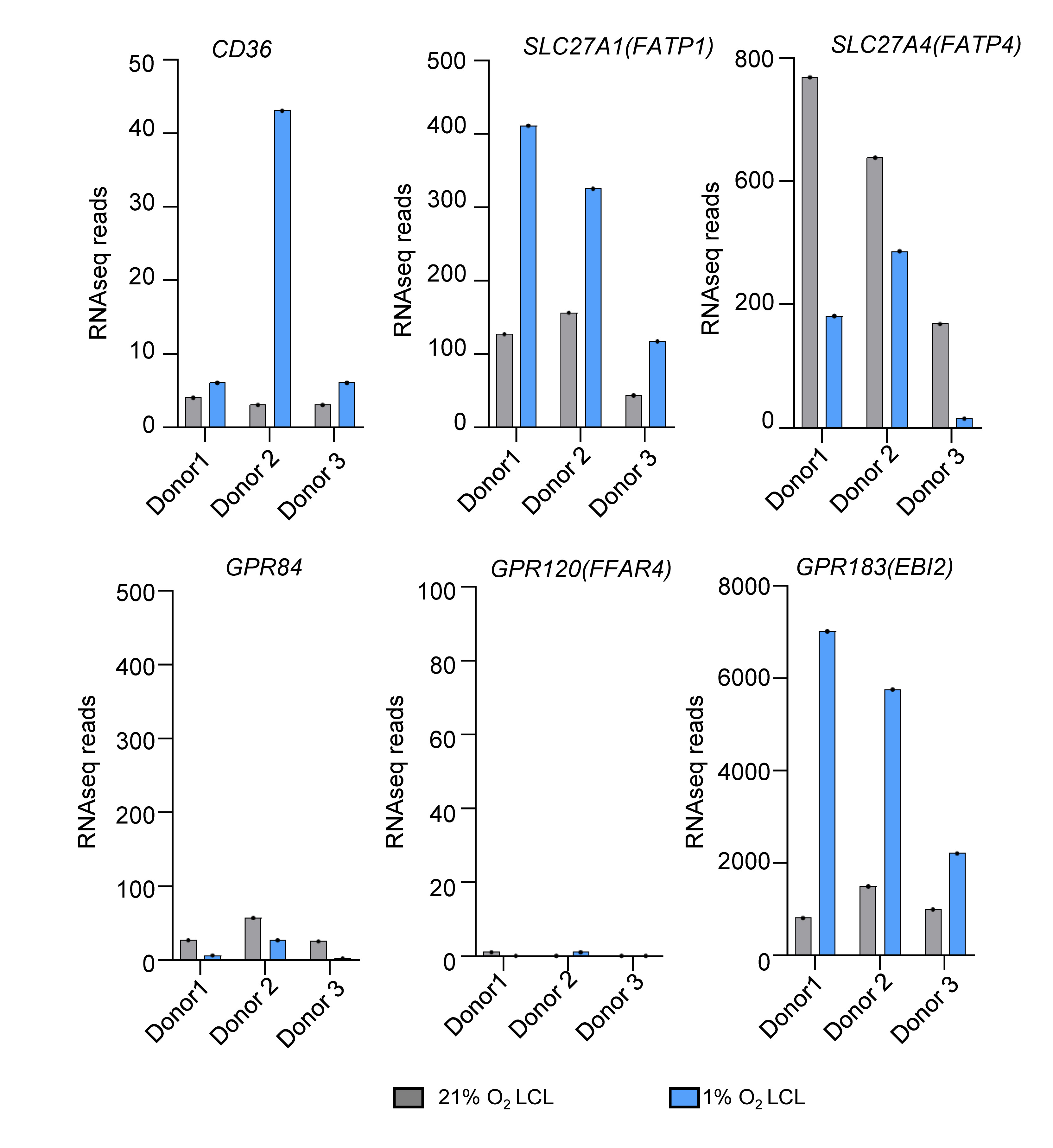

Supplement: S7 Fig — RNAseq read count of CD36, SLC27A1 (FATP1), SLC27A4 (FATP4), GPR84, GPR120 (FFAR4), GPR183 (EBI2) mRNAs in 1% or 21% O2 LCLs derived from donor 1, 2, and 3. Data is from n = 1 RNAseq replicate from independent donors. (TIF) [file ppat.1013694.s013.tif]

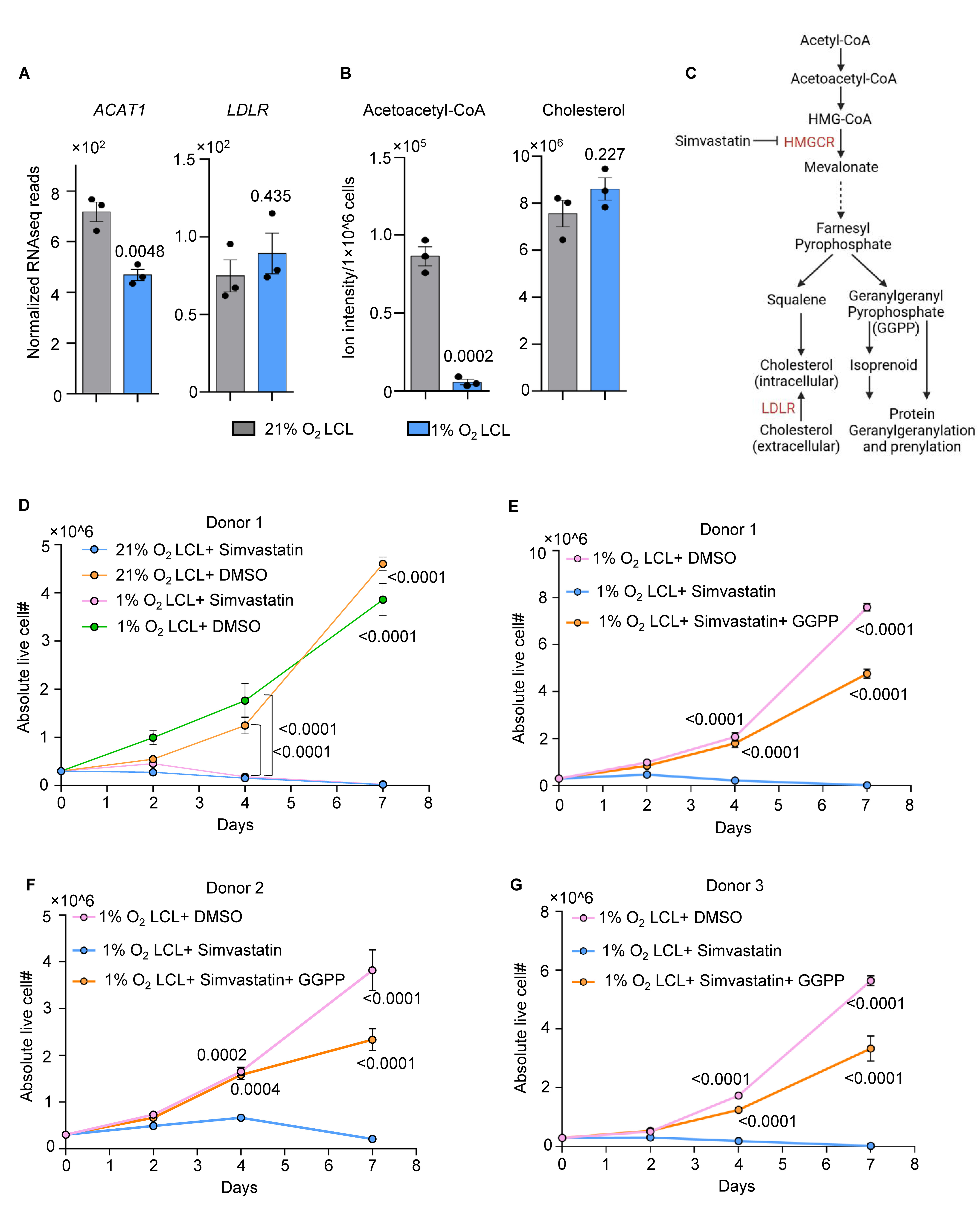

Supplement: S8 Fig — A. Bar chart analysis of DESeq2 normalized RNAseq reads of indicated genes in 1% or 21% O2 LCLs. Mean + /- SD values were from n = 3 experiments. P-values were calculated using an unpaired Student’s t-test. B. Bar chart analysis of acetoacetyl-CoA and Cholesterol ion intensity in 1% or 21% O2 LCLs. Mean + /- SD values were from n = 3 experiments using donor 1 LCLs. P-values were calculated using an unpaired Student’s t-test. C. A schematic representation of mevalonate pathway. The pathway illustrates the conversion of Acetyl-CoA to cholesterol and isoprenoids via HMG-CoA reductase (HMGCR), a key regulatory enzyme inhibited by Simvastatin. Mevalonate serves as a precursor for farnesyl pyrophosphate, which branches into cholesterol biosynthesis (via squalene) and protein prenylation (via geranylgeranyl pyrophosphate, GGPP). LDLR (low-density lipoprotein receptor) regulates extracellular cholesterol uptake. D. Growth curve of 1% or 21% O2 LCLs treated with DMSO or 2 µM Simvastatin. Cells were seeded at 3 × 10^5^/mL. Mean + /- SD values were from n = 3 experiments using donor 1 LCLs. P-values were calculated using a two-way ANOVA with Tukey’s multiple comparisons test. E-G. Growth curve of 1% O2 LCLs treated with DMSO, 2 µM Simvastatin or 2 µM Simvastatin plus 2 µM GGPP. Cells were seeded at 3 × 10^5^/mL. Mean + /- SEM values were from n = 3 experiments using LCLs derived from donor 1, 2, and 3. P-values were calculated using a two-way ANOVA with Dunnet’s multiple comparisons test, comparing each group to the 1% O₂ LCL cultured under simvastatin. (TIF) [file ppat.1013694.s014.tif]
